# 4‐Phenylbutyric Acid Reduces the Proliferation in Colon Cancer Cell Lines Through Modulating the Cell Cycle Regulatory Genes: An In Silico and In Vitro Approach

**DOI:** 10.1002/cnr2.70352

**Published:** 2025-09-15

**Authors:** Dikshita Deka, Alakesh Das, Nabajyoti Baildya, Shruthi Nagainallur Ravichandran, Surajit Pathak, Antara Banerjee, Asim K. Duttaroy

**Affiliations:** ^1^ Faculty of Allied Health Sciences Chettinad Academy of Research and Education (CARE), Chettinad Hospital and Research Institute (CHRI) Chennai India; ^2^ Department of Chemistry Milki High School Milki India; ^3^ Department of Nutrition, Institute of Basic Medical Sciences, Faculty of Medicine University of Oslo Oslo Norway

**Keywords:** 4‐phenylbutyric acid, docking, ER‐stress, intestinal inflammation, molecular dynamics simulation, unfolded protein responses

## Abstract

**Background:**

Endoplasmic reticulum stress (ER‐stress) is recognized to have a major role in both the onset and progression of various diseases, including cancer. Therefore, much research has focused on developing chemical chaperones or small compounds to reduce ER‐stress in various disease conditions.

**Aim:**

The present study investigates the effects of 4‐phenylbutyric acid (4‐PBA) on the modulation of proliferation and inflammatory responses in colon cancer cell lines by possibly regulating ER‐stress‐related protein expression.

**Materials:**

Molecular docking and molecular dynamics simulations were performed to determine the binding affinity of 4‐PBA with ER‐stress‐regulating proteins (IRE1‐α, PDI, GRP78, PERK, NRF2). To validate our hypothesis, the expression levels for ER‐stress‐regulating genes (*GRP78, XBP1, ATF6, PDI, PERK*), pro‐inflammatory genes (*CXCL12, MCP1, COX2, CCR5*), and the cell‐cycle regulatory genes (*CDK6, CCND1*), as well as the inflammatory proteins (IL‐6, IFN‐γ, and CXCL10) expression and level of catalase and ROS, were studied in colon cancer cell lines before and after treatment of different concentrations of 4‐PBA.

**Results:**

*In silico* analysis showed that 4‐PBA could bind with IRE1‐α and PERK ER‐stress proteins strongly, with the binding energy of −6.8 and −6.5 Kcal/mol. Treatment with 4‐PBA showed downregulation of pro‐inflammatory genes, along with the ER‐stress and cell‐cycle regulatory genes. The reduced expression of pro‐inflammatory proteins along with ROS and subsequent elevation in catalase levels by 4‐PBA in colon cancer cell lines indicates a correlation between ER‐stress and inflammatory response.

**Conclusion:**

The study revealed that 4‐PBA has anti‐inflammatory and anticarcinogenic properties, providing new avenues for future research.

## 1 Introduction

Endoplasmic reticulum (ER)‐stress plays a pivotal role in the pathogenesis of various disorders, including Type 2 diabetes, obesity, inflammatory bowel disease (IBD) and cancer [1, 2, 3]. Crosstalk between inflammation and unfolded protein responses (UPRs) probably influences the pathogenesis and progression of diseases involving cells linked with immune responses [1, 2, 3, 4]. Moreover, research has shown that metabolic diseases themselves can increase ER‐stress and inflammatory conditions like intestinal inflammation [5]. Metabolic disorders and intestinal inflammation form a vicious cycle, each aggravating the other. Consequently, ER‐stress‐targeted therapies focus on enhancing the clearance of misfolded proteins to reduce the ER‐stress and its inflammatory process [6, 7, 8, 9]. Small molecules that bind and promote the removal of these aberrant proteins, while modulating key pathogenic signaling pathways, have been widely investigated [10, 11]. Chemical chaperones such as 4‐phenylbutyric acid (4‐PBA) and tauroursodeoxycholic acid (TUDCA) have gained prominence for their ability to stabilize protein folding [11, 12, 13]. 4‐PBA, an aromatic fatty acid salt prodrug with anti‐inflammatory and neuroprotective properties, has been shown to reverse protein mislocalization and aggregation in various disease models [11, 12, 13, 14]. 4‐PBA has shown efficacy in diverse tumor conditions, homozygous β‐thalassemia, and spinal muscular atrophy [15], and acts as an HDAC inhibitor with ammonia‐scavenging, ER‐stress inhibition, and likely anti‐inflammatory effects [16]. Defining ER‐stress regulator gene expression and its crosstalk with inflammation in intestinal epithelial cells (IECs) could guide improved therapies for inflammatory disorders. In this study, we assessed 4‐PBA's anti‐inflammatory and anti‐cancer potential through studying the relationship with genes and proteins possibly involved in ER‐stress modulation. Notably, in a streptozotocin‐induced diabetic nephropathy rat model, 4‐PBA reduced NADPH oxidase activity, NF‐κB function, and malondialdehyde levels in kidney tissue [11, 14]. Similarly, as reported by Ono et al., treatment with 4‐PBA slowed the improvements in the condition of experimental colitis [16]. Although the actual mechanism of action was not known. In a separate investigation, 4‐PBA was chemically combined with amino acids, resulting in the production of 4‐PBA‐glutamic acid (PBA‐GA) and 4‐PBA‐aspartic acid (PBA‐AA) conjugates. The study found that PBA‐GA reduced damage and inflammation in the colon of rats with 2,4‐dinitrobenzenesulfonic acid‐induced colitis [17]. The ability of 4‐PBA to maintain cellular homeostasis by enhancing the proper folding of proteins within the ER might reduce the toxic stress response that could lead to inflammation and cellular damage [14]. In HT‐29 colon cancer cells, 4‐PBA has been shown to induce significant growth inhibition and apoptosis through the inactivation of the NF‐κB (p50:p65) heterodimer, implicating modulation of ER‐stress as a key driver of its proapoptotic effects [18]. Similarly, in HCT116 colon cancer cells treated with dehydroepiandrosterone (DHEA), co‐treatment with 1 mM 4‐PBA markedly reversed DHEA‐induced upregulation of ER‐stress markers GRP78, phosphorylated PERK, ATF4, and CHOP, concomitantly attenuating downstream apoptotic effectors DR5 and PUMA as well as the cell‐cycle arrest marker p21 [19]. Moreover, in an osthole‐treated HT‐29 model, 4‐PBA co‐administration attenuated osthole‐induced apoptosis and autophagy by suppressing ER‐stress, as evidenced by restored p62 levels and reduced LC3‐II accumulation, further underscoring the central role of ER‐stress modulation in osthole's anticancer activity [20]. Additionally, 4‐PBA has been approved by regulatory agencies like the Food and Drug Administration (FDA) for its use in treating urea cycle disorders, indicating its tolerability and safety profile for human use [14, 21]. Therefore, in the current study, we have selected 4‐PBA as our compound of interest over other available compounds. As such, understanding the role of 4‐PBA in reducing ER‐stress and its downstream effects could be pivotal for developing new strategies for research and therapy.

Caco‐2 cells are known to be derived from human colon adenocarcinoma, which might serve as an in vitro model for analyzing the inherent ER‐stress and further its associated inflammation within the context of intestinal epithelium [22]. Moreover, Caco‐2 cells spontaneously differentiate into a monolayer of polarized enterocyte‐like cells upon differentiation, closely resembling the absorptive epithelial cells of the intestinal epithelium. This cell line expresses functional brush border enzymes, exhibits tight junction formation, and develops a microvilli structure, mimicking the physiological conditions of the intestinal lining [22]. Therefore, in the present study, the Caco‐2 cell line has been further considered to study the interplay between inflammation and ER‐stress. Initially, a comprehensive evaluation of 4‐PBA was also done using both non‐cancerous (IEC‐6) and other cancerous (Caco‐2, HCT116, SW480, SW620) cell lines to ensure the effects of 4‐PBA across different cell lines.

Three primary effectors, namely pancreatic ER kinase (PKR)‐like ER kinase (PERK), activating transcription factor 6 (ATF6), and inositol‐requiring transmembrane kinase/endonuclease 1 (IRE1) of the UPR signaling pathway get activated once binding immunoglobulin protein (BiP)/glucose regulatory protein 78 (GRP78) recognizes the aggregated misfolded proteins [6, 23]. Cells persistently exposed to ER‐stress are likely to activate autophagic or apoptotic self‐destruction pathways [7]. Recent studies have shown that several tumors might require an inflammatory microenvironment, mainly because inflammation can be pro‐tumorigenic [24, 25]. Chronic inflammation drives tumorigenesis by releasing cytokines such as IL‐6, TNF, and IL‐1β, which enhance tumor cell survival, proliferation, and metastasis [25]. In the colon and small intestine of individuals with IBD and colon cancer, elevated XBP1 splicing, indicative of ER‐stress, is observed. Heazlewood et al. [8] provided evidence for the aggregation of the MUC2 precursor with cytoplasmic staining in IECs from IBD patients, as well as ultrastructural alterations in goblet cells indicative of either improper granule production or premature dissolution of retained granules before their secretion in goblet cells [8]. Furthermore, these modifications are reinforced by an increase in the levels of GRP78 in IECs, which confirms the occurrence of ER‐stress [9]. Similarly, the IRE1α‐XBP1 pathway has also been regarded as having a pro‐survival function in the UPR. Recent research has indicated that IRE1α/XBP1 plays a crucial role in maintaining malignancy in the presence of oncogenic stress [26]. Cells lacking XBP1 exhibit an inability to develop into tumor xenograft mouse models. Conversely, cells lacking XBP1 show enhanced apoptosis and reduced ability to form colonies when exposed to either ER‐stress or hypoxia [26]. Further, GRP78 is a highly active constituent of cancer cells and is excessively produced in several types of malignancies, including colon cancer [27]. GRP78 modulates cellular processes such as apoptosis, proliferation, invasion, inflammation, and immunity, particularly, in cancerous systems. Elevated levels of GRP78 in human malignancies are indicative of a greater pathological grade, increased risk of recurrence, and poor survival rates [27]. In the present study, molecular docking and molecular dynamics (MD) simulation, including RMSD, root mean square fluctuation (RMSF), SASA, and radius of gyration (*R*_g_) studies, have been performed to confirm the binding efficacy of 4‐PBA with the ER‐stress proteins such as PERK, IRE1‐α, NRF2, and PDI. Additionally, in the current study, we hypothesized that the reduction in the proliferation of colon cancer cell lines after treatment with 4‐PBA might be through the regulation of ER‐stress‐related genes.

## 2 Materials and Methods

### 2.1 In Silico Analysis: Docking of 4‐PBA With ER‐Stress Proteins

For the present study, we have selected PDI (PDB ID: 2BJX), IRE1‐α luminal domain (PDB ID: 2HZ6), NRF2 (PDB ID: 2LZ1), BiP (PDB ID: 3LDN), PERK (PDB ID: 4G34), and IRE1‐α (PDB ID: 5HGI) as ER‐stress markers. The PDB files of these selected ER‐stress markers were obtained from the Protein Data Bank (https://www.rcsb.org/). The structures of these genes were cleaned by deleting water molecules and other ligands and metals using the UCSF Chimera package. In the case of proteins containing more than one chain, only chain‐A has been considered for the study. The coordinates file for 4‐PBA was obtained from PubChem (National Library of Medicine) (CID 4775). The docking study between 4‐PBA and ER‐stress‐related proteins was performed using Autodock Vina, applying a Lamarckian genetic algorithm, and Autodock tools were used to obtain the necessary files for the docking process [28, 29].

### 2.2 Molecular Dynamics Simulation Studies of Apo and 4‐PBA Docked ER‐Stress Proteins

The minimum‐energy docked complex of 4‐PBA and the ER‐stress protein, obtained through molecular docking, was subjected to MD simulation analysis. All simulations were performed using GROMACS (version 5.1), employing the CHARMM36‐mar2019 force‐field with the TIP3P solvation model [30, 31, 32]. The CHARMM General Force Field server was used to generate the requisite input parameter files for 4‐PBA. The complex was placed in a cubic simulation box with periodic boundary conditions (PBCs) applied to emulate an infinite system and minimize boundary artifacts. The complex was positioned to maintain a minimum distance of 1 nm from the box edges to prevent interactions with its periodic images. The system was solvated with TIP3 water molecules and neutralized by adding appropriate counterions. Energy minimization was performed using the steepest descent algorithm to relieve steric clashes and bad contacts in the system. This was followed by equilibration in two phases: first under an isochoric‐isothermal (NVT) ensemble at 300 K for a duration of 100 ps, and then under an isothermal‐isobaric (NPT) ensemble at 300 K and 1 bar for 100 ps. Both equilibration phases used a time step of 2 fs. The NPT ensemble was maintained using modified Berendsen thermostats and Parrinello‐Rahman barostat.

During equilibration and production runs, a cut‐off distance of 1 nm was applied for both Van der Waals and short‐range electrostatic interactions. Long‐range electrostatics were calculated using the smooth Particle Mesh Ewald (PME) method [33]. Finally, a 20 ns production MD simulation was conducted under the NPT ensemble conditions at 300 K and 1 bar, using the same cut‐off parameters.

### 2.3 MD Simulations Study

The trajectory files were processed using the trjconv tool to remove periodic boundary artifacts and center the complex. Subsequently, structural parameters such as RMSD, RMSF, *R*_g_, and solvent‐accessible surface area (SASA) were computed using GROMACS analysis tools and visualized with XMGRACE.

## 3 In Vitro Study

### 3.1 Chemicals and Cell Line

In this study, we used IEC‐6, Caco‐2, HCT116, SW480, and SW620 cell lines, which were obtained from the National Centre for Cell Science (NCCS), Pune, India. Caco‐2, HCT116, SW480, and SW620 cells underwent 16 short tandem repeat (STR) profiling using the AmpFISTR Identifiler Plus Amplification Kit and the Applied Biosystems 3500 Genetic Analyzer (Supporting Information Data S1, supporting cell line authentication certificates). Fetal bovine serum (FBS), phosphate buffer saline (PBS), and Dulbecco's modified Eagle's medium (DMEM) were purchased from GIBCO, Thermo Fisher. Penicillin, streptomycin, amphotericin B, and trypsin were purchased from GIBCO, Thermo Fisher. DMSO and 4‐PBA (Cat No: 1821‐12‐1) were brought from Sigma‐Aldrich. Pre‐designed primers were purchased from Sigma‐Aldrich.

### 3.2 Cell Culture

All the cells were maintained at 37°C under a humidified atmosphere containing 5% CO_2_ in DMEM supplemented with FBS (10%), 100 IU/mL penicillin, and 100 μg/mL streptomycin. For the present study, a late confluent state of the cells (approximately 80% confluent) was used for all the experimental procedures.

### 3.3 Dose Determination of 4‐PBA and Cell Viability Analysis

MTT assay was performed for dose fixation of 4‐PBA in both non‐cancerous (IEC‐6) and cancerous cell lines (Caco‐2, SW480, SW620 and HCT116) to ensure accurate determination of effective dosages of 4‐PBA across different cell lines. 2 × 10^3^ per well of IEC‐6, Caco‐2, SW480, SW620, and HCT116 cells were seeded in a 96‐well plate and incubated for 24 h. Following the incubation period, the cells were exposed to different doses of 4‐PBA (25 μM, 50 μM, 100 μM, 250 μM, 500 μM, 750 μM, 1 mM, 1.5 mM, 2 mM) for a duration of 72 h. Post incubation, the cells were added with MTT solution and incubated at 37°C for 4 h, and the absorbance was measured at 546 nm using an ELISA reader.

### 3.4 Cytotoxicity Analysis

CCK‐8 assay was used to investigate the cytotoxicity of 4‐PBA on IEC‐6, Caco‐2, SW480, SW620, and HCT116 cell lines. This initial broad analysis ensured a thorough evaluation of 4‐PBA's safety and efficacy. Approximately 2 × 10^3^ cells were cultured in a 96‐well plate. Post 24 h of incubation, the cells were treated with various concentrations of 4‐PBA (100 μM, 250 μM, 500 μM, 750 μM, 1 mM, and 1.5 mM) followed by 72 h of incubation. After 72 h, CCK‐8 reagent was added according to the manufacturer's protocol, and the optical density was measured at 450 nm.

### 3.5 Expression of ER‐Stress Genes in Colon Cancer Cell Lines

RNAzol (Cat. No‐R4533) was used to isolate the RNA from the treated cells. The isolated RNA sample was quantified using Nanodrop to determine the concentration of the total RNA in each sample at 260 and 280 nm. A reverse transcription kit (Eurogentec reverse transcription kit, Belgium) was used for reverse transcription. The Syber Green qPCR master mix (Takara Bio's; Cat No: RR420A) was used to perform the qPCR using the cDNA samples. Gene expression analysis was conducted for ER‐stress genes (*XBP1, ATF 6, GRP78, PDI, PERK*) in the cancer cell lines SW480, SW620, Caco‐2, and HCT116. The *C*_t_ value of the genes, along with Δ*C*_t_ and ΔΔ*C*_t_ values, has been calculated after normalizing with the housekeeping gene *GAPDH*. The fold change was determined by calculating the relative quantification (RQ) values (2^−ΔΔ*C*t^), and the graph was plotted using GraphPad V8.4.2 software.

### 3.6 Assessment of Catalase Activity

The catalase assay was utilized to analyze the antioxidant properties of 4‐PBA in both non‐cancerous (IEC‐6) and cancerous (Caco‐2) cell lines, ensuring a comprehensive evaluation of its effects across different cellular environments. This initial assessment was critical for understanding the overall antioxidant capacity of 4‐PBA. However, further detailed analyses were solely performed on the cancerous cell line to concentrate on the potential therapeutic benefits and mechanisms of 4‐PBA specifically in the context of cancer, providing more relevant and focused insights for cancer research and treatment.

Catalase activity was analyzed using the spectrophotometric method described by Hadwan et al. with slight modifications [34]. Briefly, a 3 mL reaction mixture was prepared by adding phosphate buffer (50 mM), H_2_O_2_ (15 mM), and 0.1 mL sample. The OD was measured at 240 nm for an interval of 60 s for 3 min.

Thereafter, subsequent experimental studies were conducted in Caco‐2 cells.

### 3.7 Expression Analysis of Inflammation and Cell Cycle Regulatory Genes in the Caco‐2 Cell Line

In addition to the ER‐stress‐related genes, the analysis of the inflammatory (*CXCL12, IL‐8, COX2, MCP1, CDH1, COL1A1*, and *CCR5*) and cell proliferation (*CCND1, CDK6*) panel genes was solely performed on Caco‐2 cells. This focused approach allowed for a detailed understanding of ER‐stress responses across multiple cancer cell lines to support our hypothesis, while the genes analyzed in Caco‐2 cells provided specific insights into the interplay between inflammation and cell proliferation after 4‐PBA treatment.

### 3.8 Assessment of Expression of IL‐6, IFN‐γ, and CXCL10 Proteins in Caco‐2 Cell Line

The proteins isolated from the cell lysate were used for the assessment of the inflammatory markers like IL‐6, IFN‐γ, and CXCL10, which was done by utilizing the IL‐6 ELISA kit (Abbkine Cat No. KET6017), IFN‐γ ELISA kit (Abbkine Cat No. KET6011), and CXCL10 ELISA kit (Abbkine Cat. No‐KTE62958) according to the manufacturer's protocol.

### 3.9 Assessment of Reactive Oxygen Species (ROS) in Caco‐2 Cell Line

Dichloro‐dihydro‐fluorescein diacetate (DCFH‐DA) fluorometric assay was used to measure the intracellular ROS fluorescence. Cells were seeded in a 24‐well plate and treated with 4‐PBA for 72 h. Post treatment, the cells were stained for 30 min in the dark with 10 μM DCFH‐DA. Further, to stain the nucleus, Hoechst stain at a concentration of 1 μg/mL was used and incubated for 10 min in the dark. The fluorescence intensity was measured after a single PBS wash using a fluorescence microscope, and further, it was quantified using ImageJ software.

### 3.10 β‐Galactosidase (SA‐β‐Gal) Assay for Senescence Assessment in the Caco‐2 Cell Line

The EZdetect cell senescence detection kit was used to measure the SA‐β‐Gal cell senescence activity using a chromogenic substrate (HiMedia Cat. No‐CCK063). The staining was performed according to the manufacturer's protocol. The stained cells were observed, and images were taken using an inverted light microscope.

### 3.11 Statistical Analysis

Statistical analysis was performed by using GraphPad V8.4.2 software (online version). The significant difference between the treatment and control groups was evaluated by the student's t‐test. The results are presented as mean ± standard error. The asterisks *, **, and *** indicate the *p*‐values of 0.05, 0.01, and 0.001, respectively, which were regarded as statistically significant, and ns = nonsignificant. All the experiments were performed in triplicate.

## 4 Results

### 4.1 Docking of 4‐PBA With ER‐Stress Markers

**FIGURE 1:**
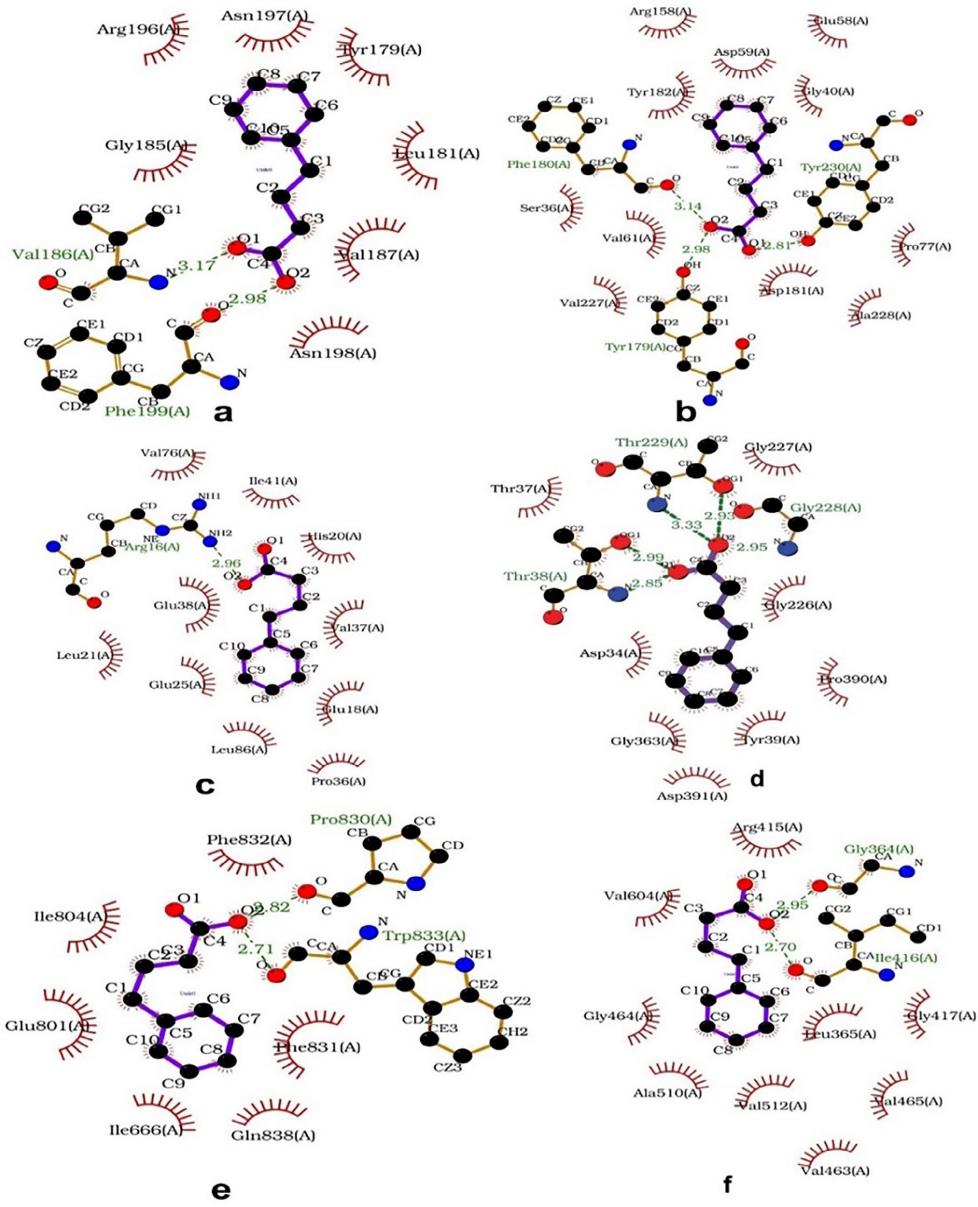
2D contour plot of 4‐PBA docked into the cavity of ER‐stress markers. (a) 2BJX, (b) 2BZ6, (c) 2LZ1, (d) 3LDN, (e) 4G34, and (f) 5HGI.

Molecular docking suggests that the IRE1‐α luminal domain (PDB ID: 2HZ6) has the highest binding affinity (−6.8 Kcal/mol) with 4‐PBA, as shown in Table 1 with its nearest residues. The inhibition constant value also revealed that 4‐PBA binds strongly in the cavity of IRE1‐α and PERK, ER‐stress markers. Figure 1 represents a 2D contour plot between 4‐PBA and the ER‐stress markers, where the exact binding cavity of 4‐PBA with the nearest residue with a distance of 5 Å is shown. Further, the negative control residues of ER‐stress markers with 4‐PBA are displayed in Table S1.

**TABLE 1:** Binding affinity of 4‐PBA against different ER‐stress markers with corresponding inhibition constant values and nearest residues.

ER‐stress marker	PDB‐ID	Binding affinity (Kcal/mol)	Inhibition cont. (*K*_i_) (μM)	Nearest residues	3D‐binding cavity
PDI	2BJX	−5.3	130.55	Tyr179, Leu181, Gly185, Val186, Val187, Arg196, Asn197, Asn198, Phe199	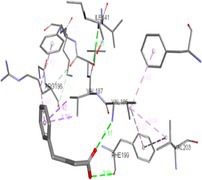
IRE1‐α luminal domain	2HZ6	−6.8	10.39	Ser36, Gly40, Glu58, Asp59, Val61, Pro77, Arg158, Tyr179, Phe180, Asp181, Val227, Ala228, Tyr230	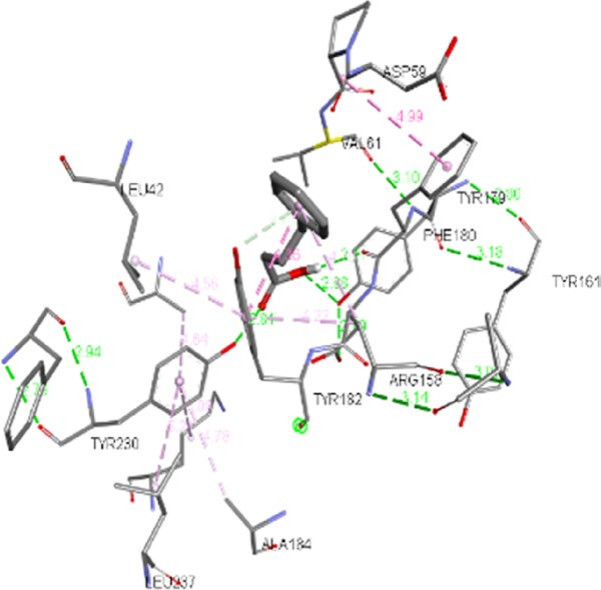
NRF2	2LZ1	−5.7	66.47	Arg16, Glu18, His20, Leu21, Glu25, Val37, Glu38, Ile41, Leu86, Val76	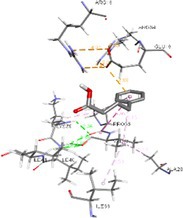
GRP78	3LDN	−6.1	33.84	Asp34, Thr37, Thr38, Tyr39, Gly226, Gly227, Gly228, Thr229, Gly363, Pro390, Asp391	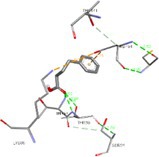
PERK	4G34	−6.5	17.23	Leu598, Val606, Val651, Met887, Cys890, Phe943, Gly953, Asp954	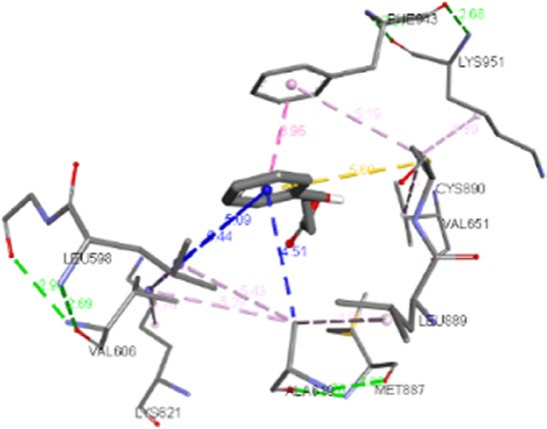
IRE1‐α	5HGI	−5.9	47.43	Ile666, Glu801, Ile804, Pro830, Phe831, Phe832, Trp833, Gln838	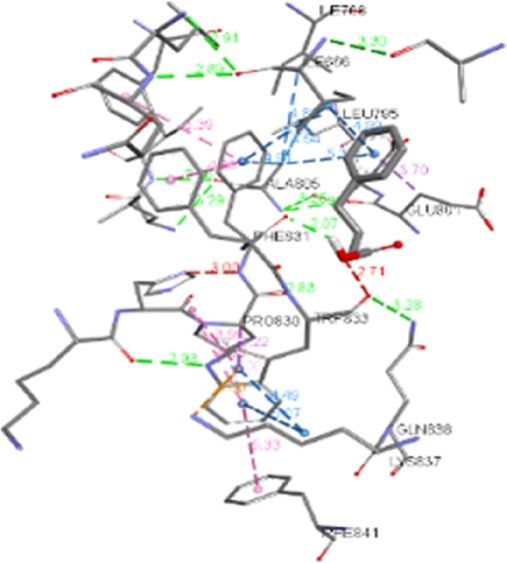

### 4.2 MD Simulation Studies of Apo and 4‐PBA Docked ER‐Stress Markers

**FIGURE 2:**
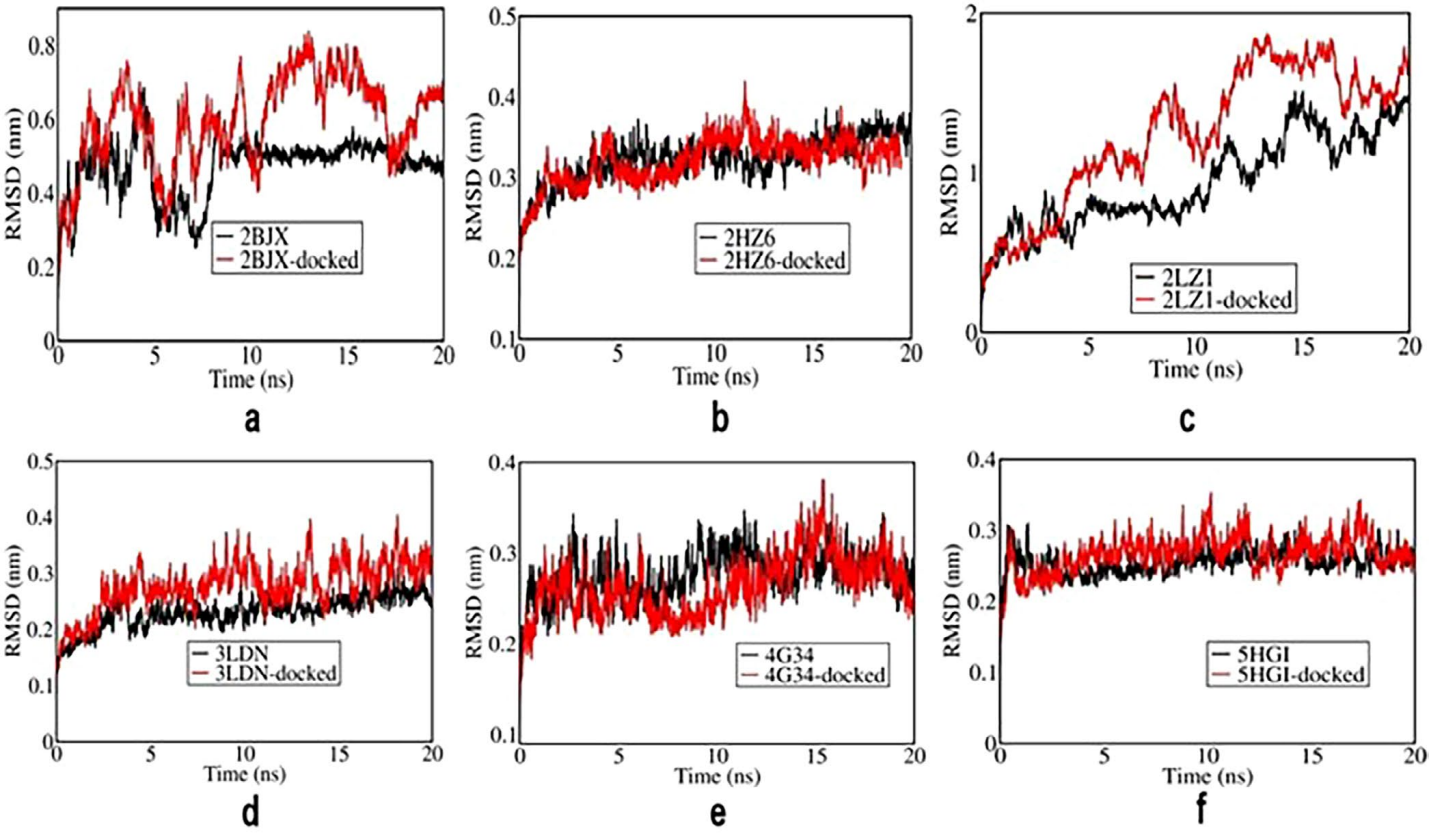
(a–f) RMSD plots of 4‐PBA‐docked and apo stress markers where 2BJX is PDI, 2HZ6 is IRE1α (luminal domain), 2LZ1 is NRF2, 3LDN is GRP78, 4G34 is PERK, and 5HG1 is IRE1α ER‐stress proteins.

As depicted in Figure 2, RMSD analysis was performed to assess the structural stability of the apo (black) and 4‐PBA‐docked (red) ER‐stress protein complexes during a 20 ns MD simulation. Figure 2a represents the RMSD plot between 4‐PBA and PDI. It is clear from the figure that after 10 ns, the apo‐PDI has reached an equilibration indicated by lesser fluctuation, while the equilibration is disturbed due to the presence of 4‐PBA. Figure 2b indicates the RMSD plot between 4‐PBA and IRE1‐α. Here, 4‐PBA stabilizes the structure of ER‐stress proteins after 1 ns and is maintained throughout the simulation process. Figure 2c shows the RMSD plot of NRF2 (2LZI), where both the apo and 4‐PBA‐bound forms exhibit substantial fluctuations. However, the 4‐PBA‐bound form shows consistently higher deviations, suggesting that 4‐PBA binding may increase the conformational flexibility of NRF2 during the simulation. As shown in Figure 2d–f, the RMSD profiles of 3LDN, 4G34, and 5HGI complexes indicate that the systems reached equilibration, with minor fluctuations throughout the 20 ns simulation. This suggests that the binding of 4‐PBA did not significantly destabilize these proteins.

**FIGURE 3:**
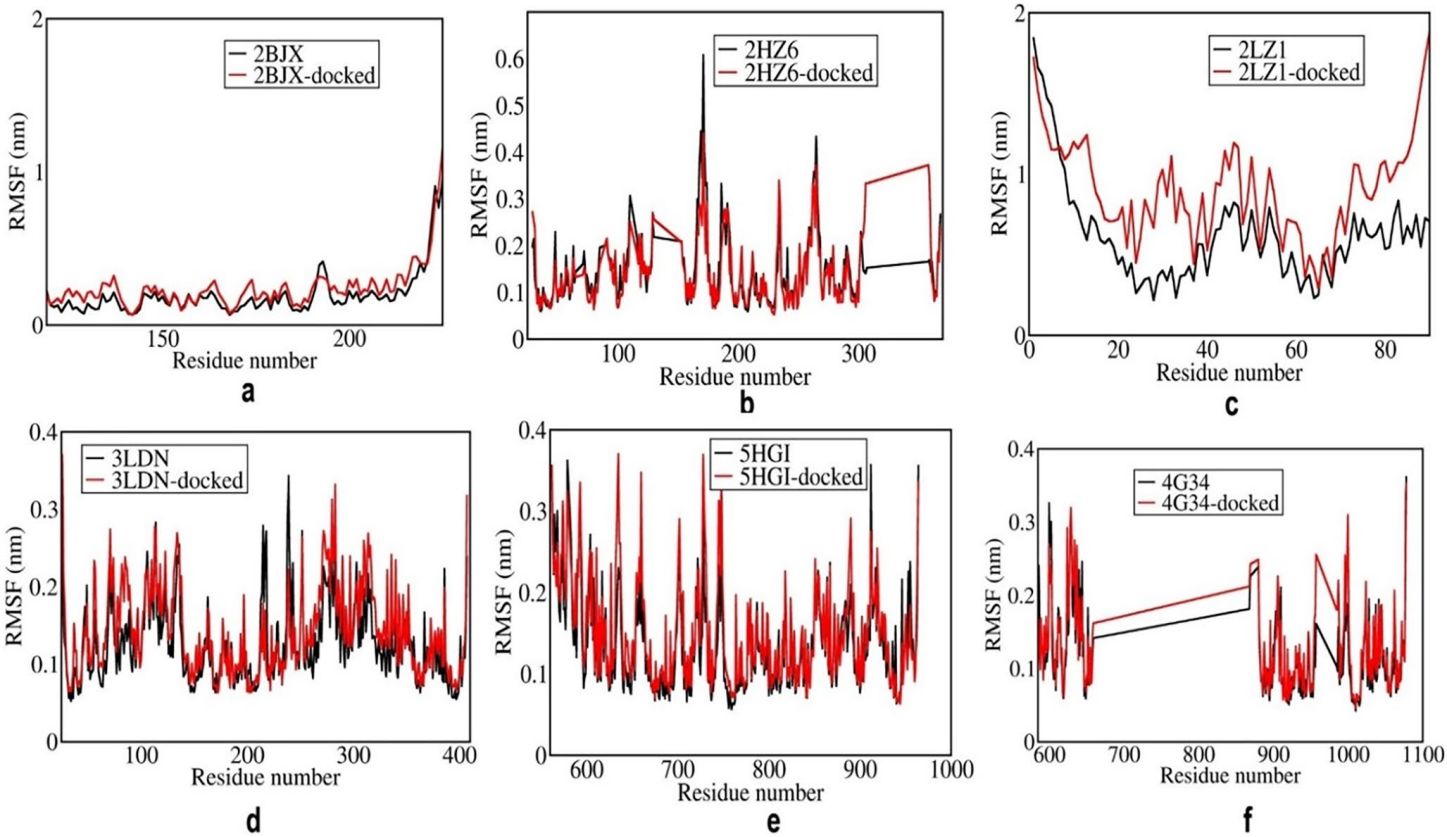
(a–f) RMSF plots of 4‐PBA‐docked and apo ER‐stress markers where, 2BJX is PDI, 2HZ6 is IRE1α (luminal domain), 2LZ1 is NRF2, 3LDN is GRP78, 4G34 is PERK, and 5HG1 is IRE1α ER‐stress proteins.

Figure 3a–f represents the RMSF curve for the ER‐stress proteins in apo (black) and docked form with 4‐PBA (red), highlighting residue‐level fluctuations over the simulation period. The figure demonstrates that upon binding with 4‐PBA, the ER‐stress proteins such as 1RE‐1α and PERK exhibit increased flexibility at specific residues, including ARG, GLU, LEU, ARG, TYR, and PHE. These localized fluctuations of residues suggest that 4‐PBA induces conformational changes in the docked structures compared to their apo forms. These observations indicate potential interaction between 4‐PBA and the IRE1‐α and PERK stress proteins, which are further supported by the corresponding RMSD data.

**FIGURE 4:**
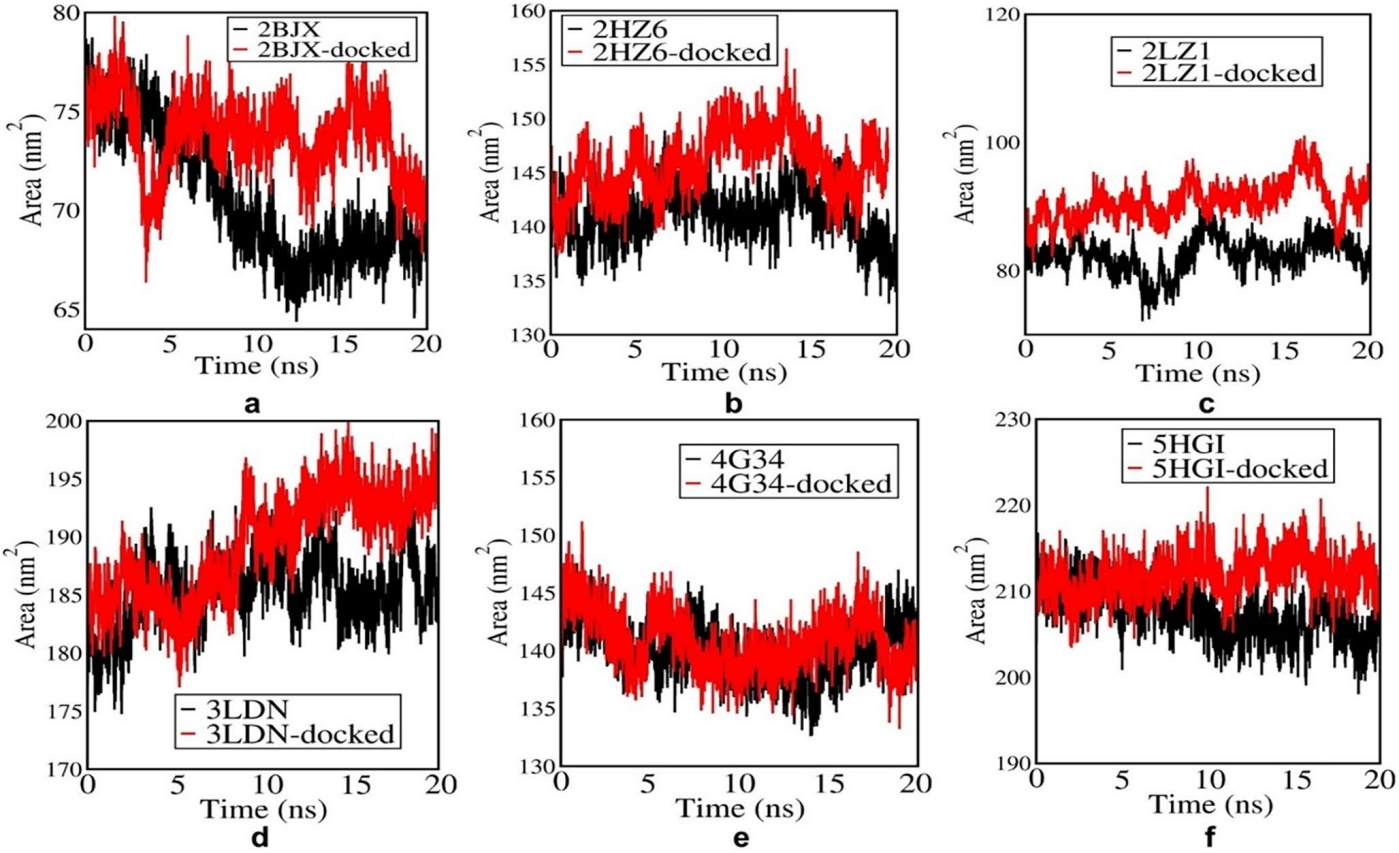
(a–f) SASA plots of 4‐PBA‐docked and apo ER‐stress markers where, 2BJX is PDI, 2HZ6 is IRE1α (luminal domain), 2LZ1 is NRF2, 3LDN is GRP78, 4G34 is PERK, and 5HG1 is IRE1α ER‐stress proteins.

Figure 4 illustrates the SASA profiles of apo (black) and 4‐PBA‐docked (red) ER‐stress proteins over a 20 ns MD simulation. In Figure 4a (2BJX‐PDI), the docked protein exhibits a consistently higher SASA with notable fluctuations, indicating increased solvent exposure likely due to ligand‐induced conformational changes or partial unfolding compared to its apo counterpart. Figure 4b (2HZ6‐IRE1‐α) shows a higher SASA for the docked complex compared to the apo form, with relatively lower fluctuations, suggesting a more stabilized yet expanded conformation upon 4‐PBA binding. In Figure 4c (2LZI‐NRF2), the SASA of the docked structure remains consistently higher than that of the apo form throughout the simulation, indicating enhanced surface exposure and structural rearrangement induced by ligand binding. In Figure 4d (3LDN), this increase is accompanied by noticeable fluctuations, suggesting dynamic rearrangements at the surface level post‐docking. However, in Figure 4e (4G34), although the docked form also displays a higher SASA on average, the overall fluctuation range remains comparable or slightly less than the apo form, indicating a marginally more exposed but relatively stable conformation rather than high dynamic surface activity. In Figure 4f (5HGI‐IRE‐α), the docked form shows consistently higher SASA with minimal fluctuations, indicating a stable conformation over time.

**FIGURE 5:**
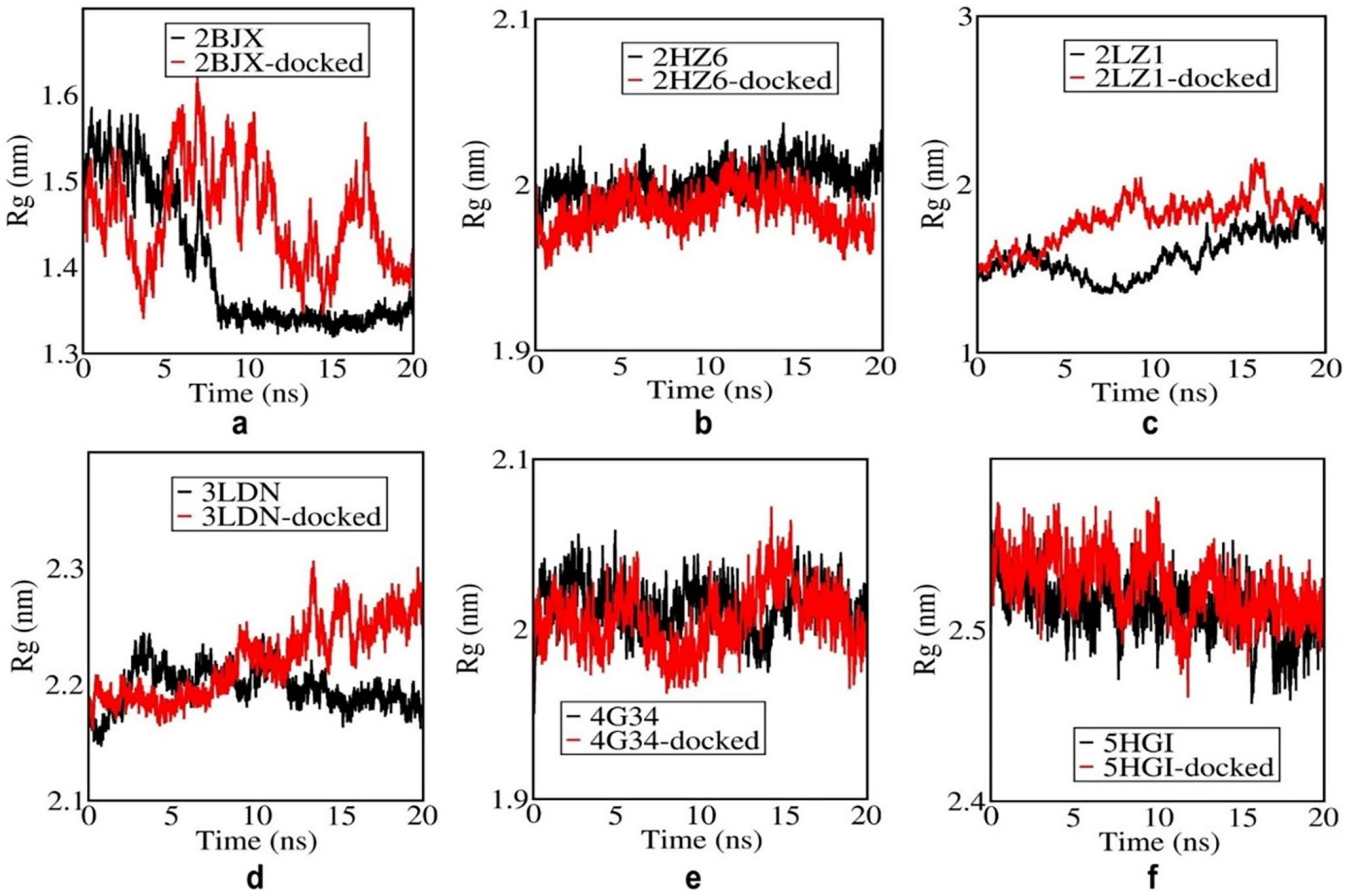
(a–f) Radius of gyration plots of 4‐PBA‐docked and apo ER‐stress markers where, 2BJX is PDI, 2HZ6 is IRE1α (luminal domain), 2LZ1 is NRF2, 3LDN is GRP78, 4G34 is PERK, and 5HG1 is IRE1α ER‐stress proteins.

The *R*_g_ reflects the compactness of a protein system during simulation. A lower *R*_g_ value indicates a more compact structure, while a higher *R*_g_ value indicates a more expanded or less compact conformation. Figure 5a–f shows the *R*_g_ plots for the apo (black) and 4‐PBA‐docked (red) ER‐stress proteins. It can be seen that the apo forms generally exhibit lower *R*_g_ values, indicating a more compact structure compared to the docked forms. However, Figure 5b shows an exception where the apo form has a higher *R*_g_, suggesting it adopts a less compact conformation, which may reflect conformational changes or partial unfolding during simulation. These changes indicate the dynamic behavior and stability differences of the ER‐stress proteins upon ligand binding during the MD simulation.

### 4.3 Dose Determination of 4‐PBA and Cell Viability Analysis by MTT Assay

**FIGURE 6:**
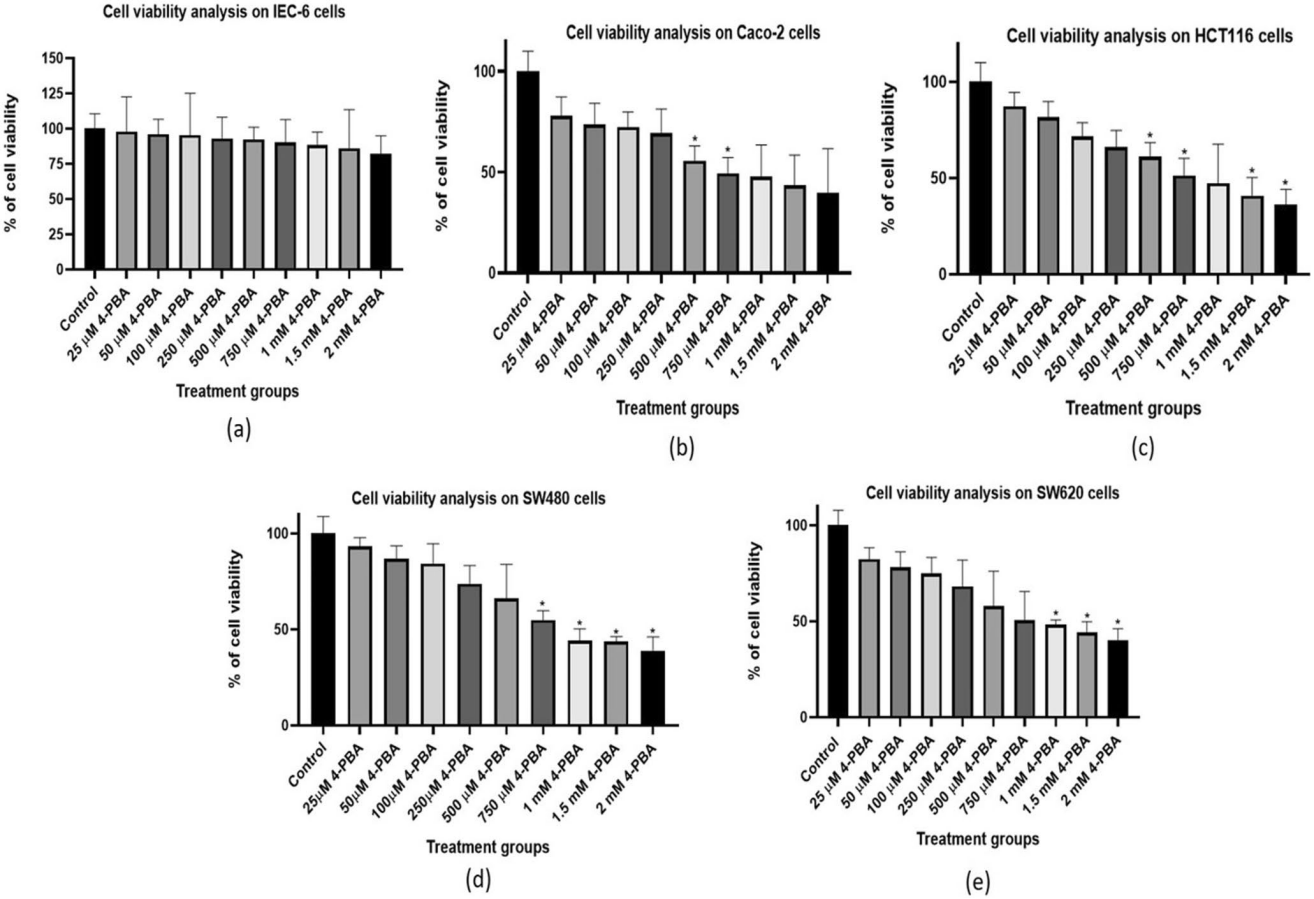
The graph represents the viability of cells in various cell lines by MTT assay with various concentrations of 4‐PBA. (a) Cell viability analysis on IEC‐6 cells, (b) cell viability analysis on Caco‐2 cells, (c) cell viability analysis on HCT116 cells, (d) cell viability analysis on SW480 cells, and (e) cell viability analysis on SW620 cells. The statistical significance is represented as *p < 0.05.

The non‐cancerous and cancerous cells were treated with various concentrations (25 μM, 50 μM, 100 μM, 250 μM, 500 μM, 750 μM, 1 mM, 1.5 mM, 2 mM) of 4‐PBA. The results revealed a notable dose‐dependent decrease in cell viability among colon cancer cells (Caco‐2, SW480, SW620, and HCT116) treated with 4‐PBA as compared to the untreated group (control) (Figure 6b–e). Conversely, the changes observed in non‐cancerous cells (IEC‐6) treated with 4‐PBA were not significantly altered when compared to the untreated control group of IEC‐6 cells (Figure 6a), indicating the ability of 4‐PBA to induce cytotoxicity specifically in colon cancer cells while minimizing adverse effects on non‐cancerous cells (Figure 6a). Hence, the doses of 500 μM, 750 μM, and 1 mM were selected for further study given that the lower doses did not show notable differences and the higher doses were more lethal for the selected cell line.

### 4.4 Cytotoxicity Analysis

**FIGURE 7:**
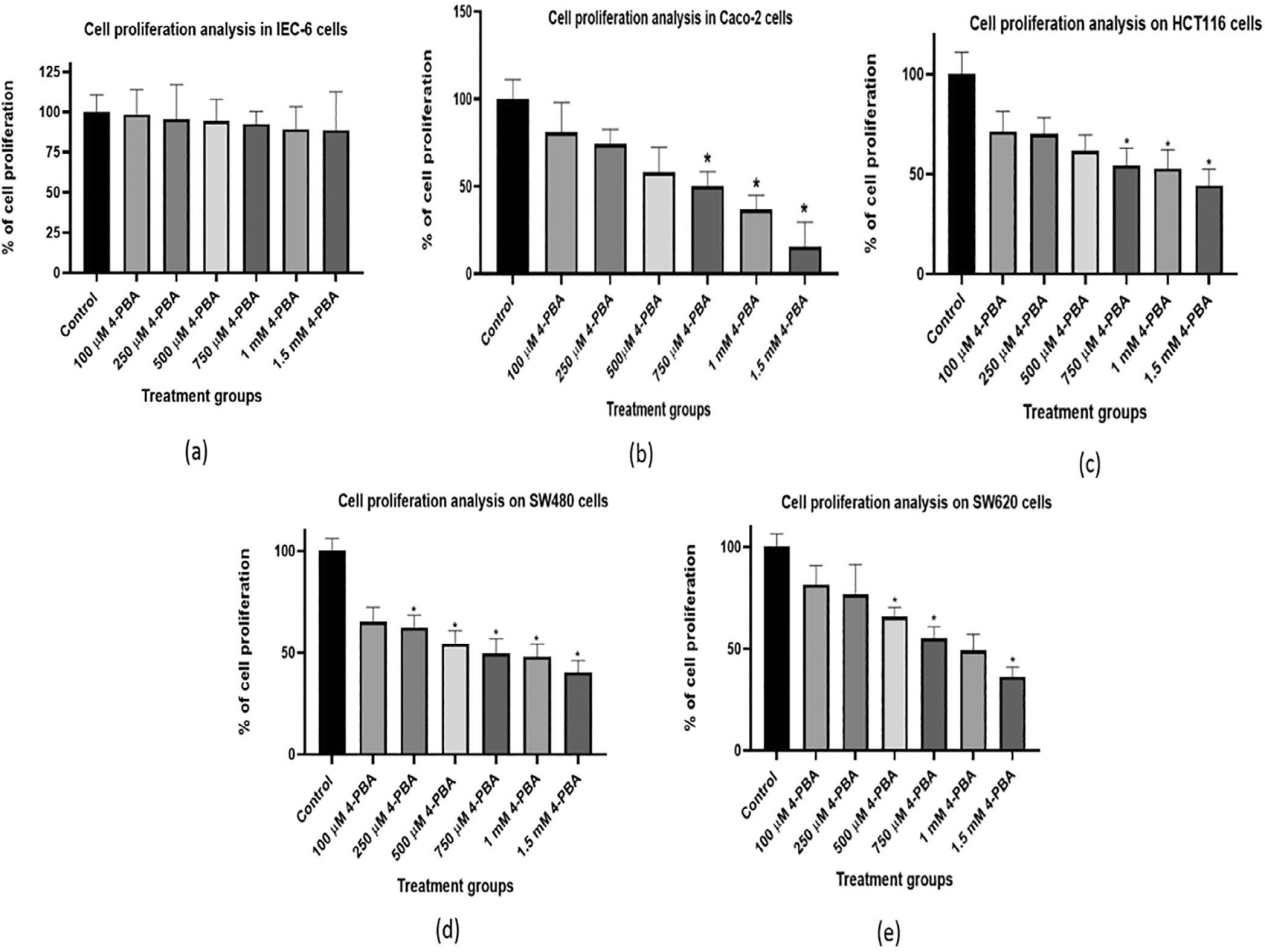
The graph represents the proliferation of cells by CCK‐8 assay with various concentrations of 4‐PBA. (a) Cell proliferation analysis in IEC‐6 cells, (b) cell proliferation analysis in Caco‐2 cells, (c) cell proliferation analysis in HCT116 cells, (d) cell proliferation analysis in SW480 cells, and (e) cell proliferation analysis in SW620 cells. The statistical significance is represented as *p < 0.05.

CCK‐8 assay results showed an increase in the cytotoxicity of 4‐PBA in the 4‐PBA‐treated colon cancer cells (100 μM, 250 μM, 500 μM, 750 μM, 1 mM, and 1.5 mM), which was measured as per the proliferation of the cells compared to the untreated colon cancer cells in a dose‐dependent manner after 72 h of treatment (Figure 7b–e). Similarly, in line with the MTT assay, the changes in the 4‐PBA‐treated IEC‐6 cells showed minimal cytotoxicity. Therefore, the CCK‐8 assay validated the cytotoxic property of 4‐PBA in colon cancer cells; however, in non‐cancerous cells (IEC‐6), 4‐PBA treatment was not found to be significantly altered (Figure 7a).

### 4.5 Expression Analysis of ER‐Stress‐Related Genes in Colon Cancer Cell Lines

**FIGURE 8:**
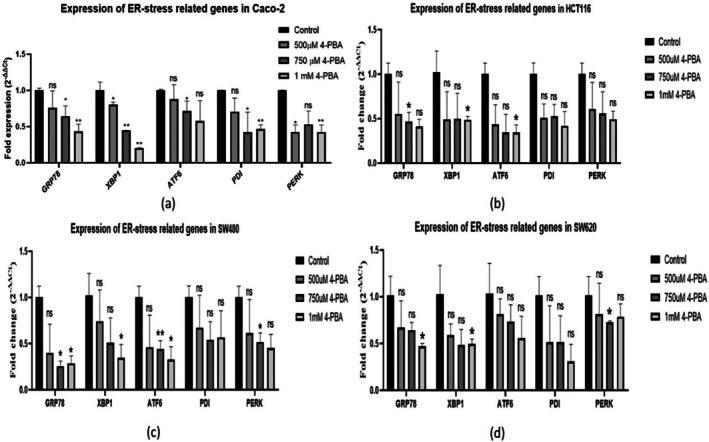
Graph representing the fold expression of ER‐stress‐related genes in control and 4‐PBA‐treated colon cancer cell lines. (a) Expression of ER‐stress‐related genes in Caco‐2, (b) expression of ER‐stress‐related genes in HCT116, (c) expression of ER‐stress‐related genes in SW480, and (d) expression of ER‐stress‐related genes in SW620. The statistical significance is represented as *p < 0.05; **p < 0.01; ***p < 0.001, and ns = non‐significant.

The expression of the ER‐stress‐associated genes was performed in colon cancer cell lines (Caco‐2, SW480, SW620 and HCT116), and the results showed that 4‐PBA was potent in reducing the expression levels of the ER‐stress markers *GRP78, XBP1, PERK, PDI*, and *ATF6*, as shown in (Figure 8a–d), signifying that 4‐PBA might be a potent candidate in resolving the misfolded or unfolded proteins.

### 4.6 Catalase Activity

**FIGURE 9:**
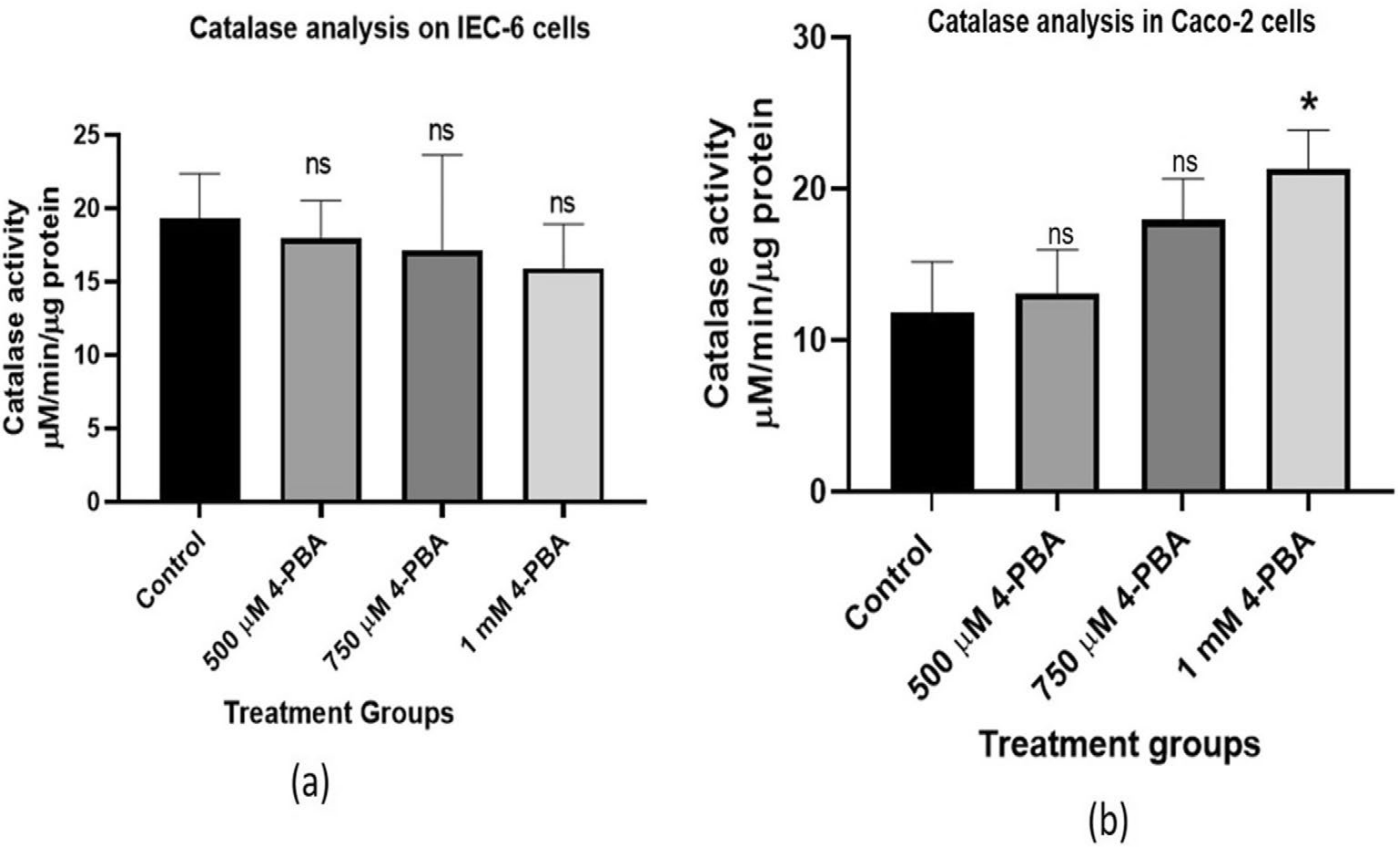
Catalase activity was assessed to evaluate the impact of 4‐PBA on Caco‐2 cells. The bar graph represents the activity of catalase in the control and 4‐PBA‐treated groups. (a) Catalase analysis on IEC‐6 cells and (b) catalase analysis on IEC‐6 cells. The statistical significance is represented as *p < 0.05 and ns = non‐significant.

The non‐cancerous cells (IEC‐6) treated with 4‐PBA showed minimal alterations in the level of catalase as compared to the untreated control cells; contrary to untreated Caco‐2 cells, a substantial decline in catalase activity was seen, pointing to the depletion of antioxidant enzymes. Treatment with 4‐PBA at a concentration of 1 mM to the Caco‐2 cells was observed to significantly restore the initial level of catalase activity (Figure 9).

### 4.7 Expression Analysis of Inflammation‐Related and Cell Cycle Regulatory Genes in the Caco‐2 Cell Line

#### 4.7.1 Inflammation‐Regulatory Gene Expression Analysis

**FIGURE 10:**
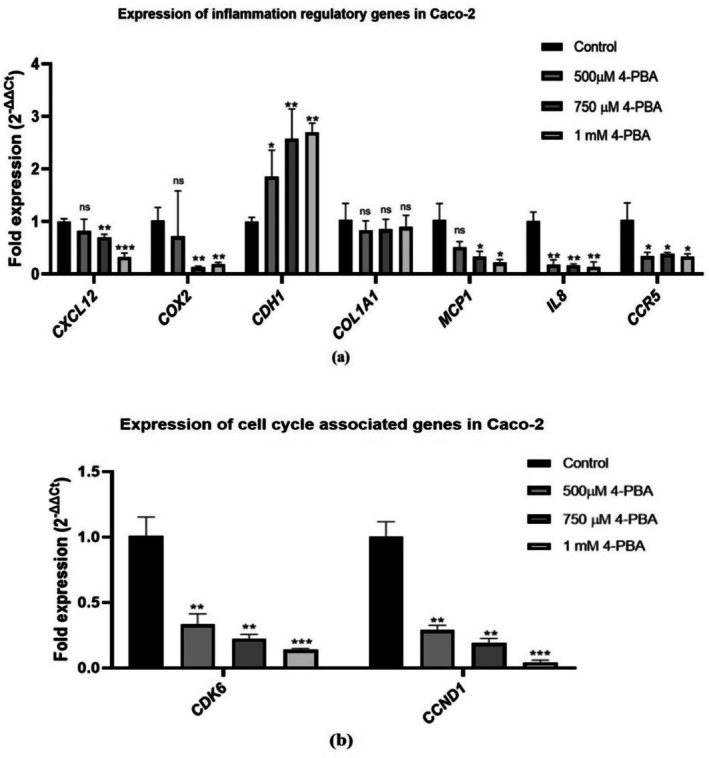
Graph representing the fold expression of inflammation‐regulatory markers and cell cycle regulatory genes in control and 4‐PBA‐treated Caco‐2 groups. (a) Expression of inflammation regulatory genes in Caco‐2 and (b) expression of cell cycle associated genes in Caco‐2. The statistical significance is represented as *p < 0.05; **p < 0.01; ***p < 0.001; and ns = non‐significant.

Likewise, the efficacy of 4‐PBA in the inflammatory genes *CXCL12, IL‐8, CCR5, CDH1, MCP1, COX2*, and *COL1A1* was assessed as their expression patterns affect the disease development and severity. After being treated with the selected concentrations of 4‐PBA, *CDH1* expression was observed to be elevated, whereas the expression of pro‐inflammatory genes *CXCL12, IL‐8, CCR5, MCP1*, and *COX2* was found to be downregulated significantly when compared with the control group. However, *COL1A1* did not show any significant changes in the treated groups (Figure 10a).

#### 4.7.2 Cell Proliferation Regulatory Gene Expression Analysis

To determine the proliferation‐related gene expression after treating Caco‐2 cells with selected concentrations of 4‐PBA, the expression of proliferation‐associated genes Cyclin D1 (*CCND1*) and cyclin‐dependent kinase (*CDK6*) was studied. 500 μM, 750 μM and 1 mM treatment on Caco‐2 cells has shown a significant decrease in the expression of *CCND1* and *CDK6* as depicted in Figure 10b.

### 4.8 Expression of Inflammatory Markers IL‐6, IFN‐γ, CXCL10 in Caco‐2 Cell Line

**FIGURE 11:**
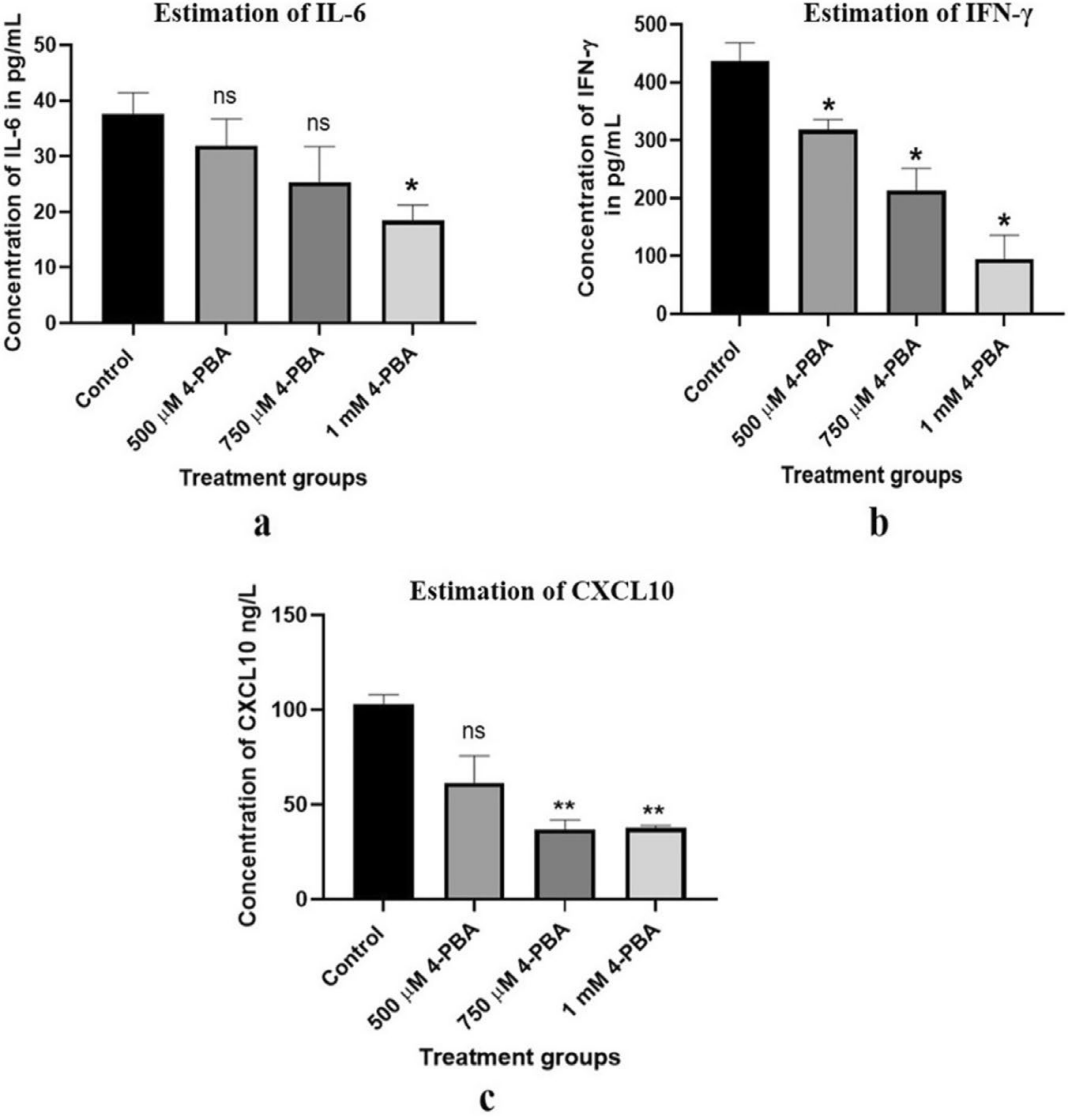
Expression of IL‐6 (a), IFN‐γ (b), and CXCL10 (c) in control and 4‐PBA treated groups. The statistical significance is represented as *p < 0.05; **p < 0.01; and ns = non‐significant.

For the present analysis, Caco‐2 cells treated with a 1 mM concentration of 4‐PBA showed a significant decrease in the level of IL‐6 when compared with the control group. IFN‐γ is found to potentiate pro‐inflammatory signaling and cause inflammatory responses. Our results suggest that the selected doses of 4‐PBA could significantly reduce the expression of IFN‐γ when compared to the control groups. Likewise, the outcome from the current analysis revealed that the 4‐PBA‐treated groups have significantly reduced the expression of CXCL10 when compared with the control group (Figure 11a–c).

### 4.9 Assessment of ROS

**FIGURE 12:**
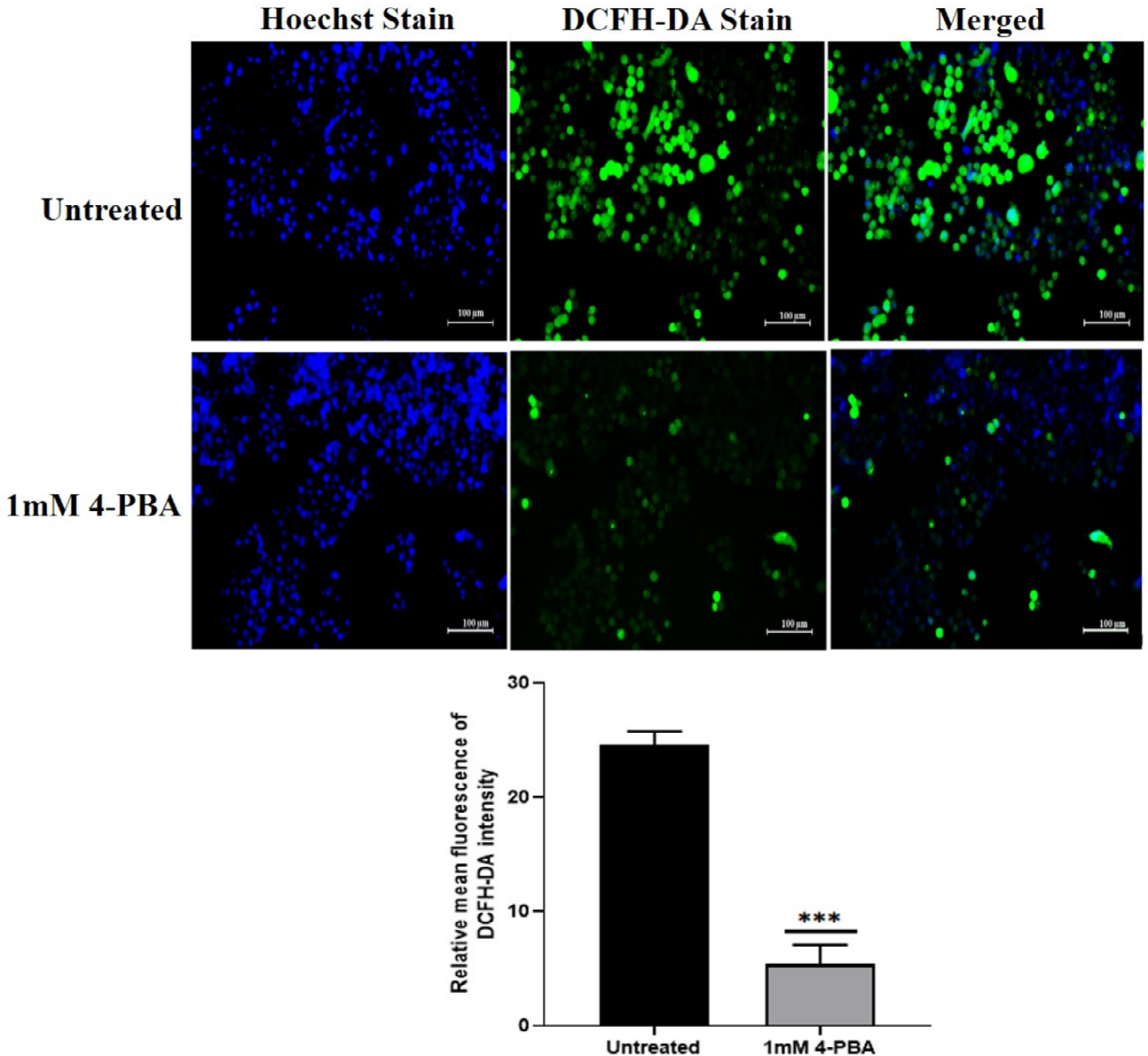
Figure showing the fluorescence intensity of DCFH‐DA stain in the untreated and 4‐PBA‐treated groups in the Caco‐2 cell line. The statistical significance is represented as ***p < 0.001.

Fluorescein‐labeled dye DCFH‐DA was used to detect the accumulation of intracellular ROS. As shown in Figure 12, the control group displayed a higher intensity of green fluorescence, whereas in the 1 mM 4‐PBA‐treated group, a distinct difference in the fluorescence intensity was observed, signifying reduced accumulation of ROS, and the fluorescence intensity was quantified with ImageJ software.

### 4.10 SA‐β‐Gal Assay for Senescence Study

**FIGURE 13:**
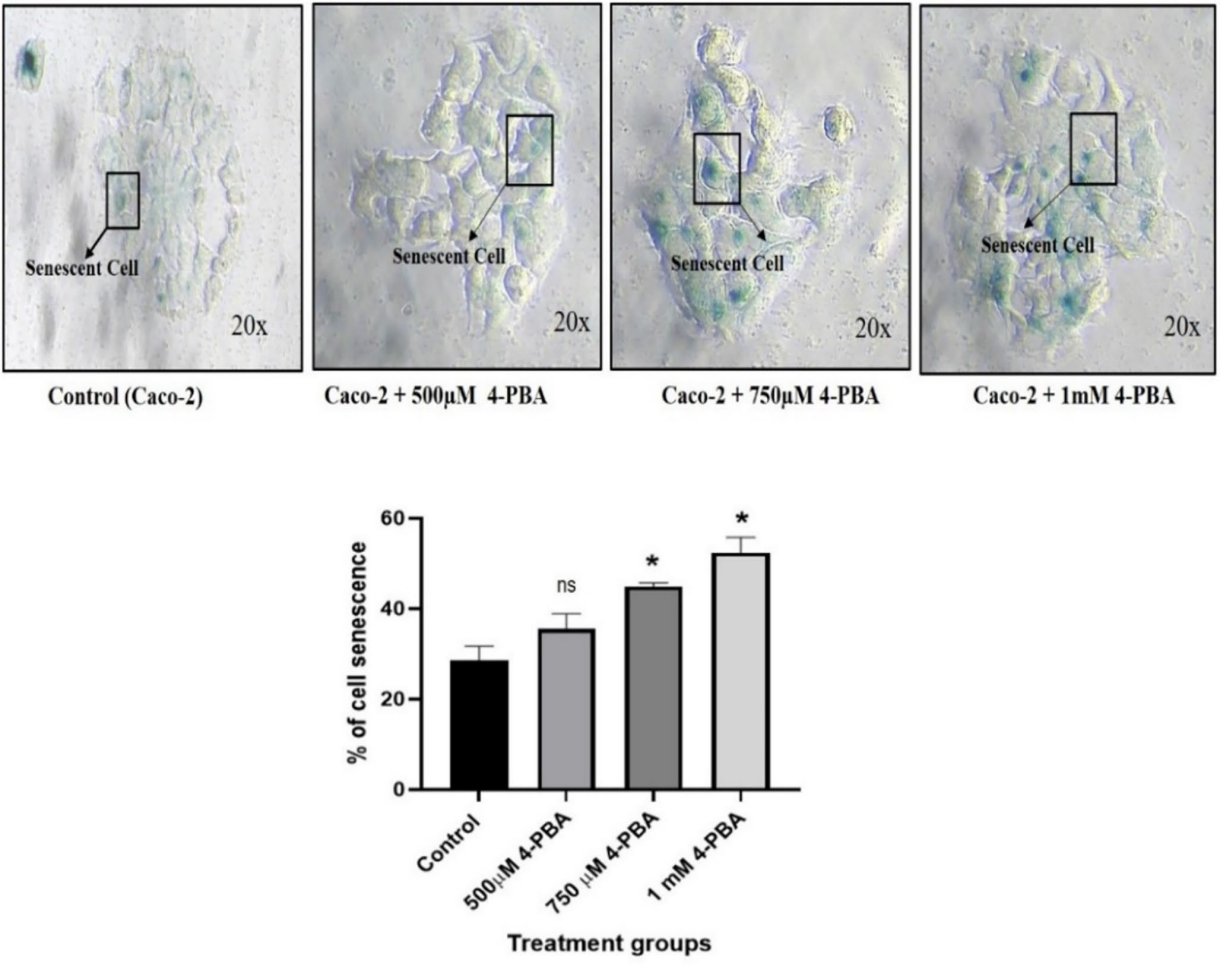
SA‐β‐Gal staining of the control group and the 4‐PBA‐treated groups with their respective concentrations (500 μM, 700 μM, and 1 mM) along with the graph representing the percentage of senescent cells in the control and treated groups. The statistical significance is represented as *p < 0.05 and ns = non‐significant.

Staining with SA‐β‐gal revealed a significant amount of senescent activity in Caco‐2 cells. When compared to the control group, the levels of senescence in 4‐PBA‐treated Caco‐2 cells were observed to be considerably increased (Figure 13). The blue deposits in the cells indicate cellular senescence. Our results suggested that 4‐PBA could induce cellular senescence in Caco‐2 cells.

## 5 Discussion

Preclinical investigations have established that 4‐PBA functions as an effective chemical chaperone, attenuating ER‐stress and dampening inflammatory signaling in colon cancer cell lines. Originally approved in the 1980s for the management of urea cycle disorders [14], 4‐PBA has since attracted considerable interest for its ability to stabilize protein folding within the ER. ROS and pro‐inflammatory stimuli are well‐known activators of ER‐stress pathways [35, 36] and persistent ER dysfunction in IECs has been implicated in the pathogenesis of both IBD and colon carcinogenesis [35, 36, 37, 38]. In vitro studies using HT‐29 and HCT116 colon cancer cell lines demonstrated that treatment with 4‐PBA not only inhibited cellular proliferation and induced apoptosis but also normalized the expression of key ER‐stress markers [39, 40].

In vivo, administration of 4‐PBA in an azoxymethane/dextran sulfate sodium (AOM/DSS) mouse model of colitis‐associated colorectal cancer led to the suppression of PDIA2 overexpression, restoration of metabolic homeostasis, and a marked reduction in tumor burden [41]. These results underscore 4‐PBA's potential as a modulatory agent against ER‐stress‐driven tumorigenesis. Nevertheless, despite its established safety profile in metabolic disorders, 4‐PBA has not yet progressed to clinical evaluation in colorectal cancer patients. To determine its viability as a therapeutic agent, early‐phase clinical trials employing UPR‐related biomarkers are warranted. Such studies would clarify optimal dosing, safety in oncology settings, and its capacity to synergize with standard chemotherapeutic regimens.

This study employed molecular docking and MD simulations to investigate the interaction between 4‐PBA and ER‐stress regulatory proteins. Results showed that 4‐PBA binds effectively to these ER‐stress proteins, especially IRE1 and PERK, suggesting an influence on their conformational change. MD simulations over 20 ns, supported by RMSD, RMSF, SASA, and *R*_g_ analyses, confirmed these conformational changes. The binding of 4‐PBA to IRE1 may affect its endoribonuclease activity and XBP1 mRNA splicing, while interaction with PERK could alter its kinase activity, impacting eIF2α phosphorylation and subsequent protein synthesis under stress conditions.

The study observed that ER‐stress proteins such as XBP1, ATF6, PERK, PDI, and BiP typically exhibit increased expression during cellular stress responses [42]. In colon cancer cell lines (Caco‐2, SW480, SW620, HCT116), overexpression of BiP and these markers reflects increased ER‐stress. Treatment with 4‐PBA led to a marked reduction in *GRP78* and other ER‐stress genes (*XBP1, ATF6, PDI, PERK*), suggesting its role in reducing ER‐stress. Additionally, 4‐PBA significantly downregulated inflammatory gene expression, including *CXCL12, CCR5, IL‐8, COX2*, and *MCP1*, key players in establishing a pro‐tumorigenic inflammatory microenvironment [43]. Conversely, the upregulation of *CDH1*, an anti‐inflammatory gene, further supports the anti‐inflammatory effect of 4‐PBA. These outcomes align with prior reports linking ER‐stress and UPR activation to conditions like ulcerative colitis [8, 44]. 4‐PBA also suppressed the expression of cell proliferation genes *CCND1* and *CDK6*, which are critical for cell cycle progression. This downregulation may hinder tumor growth by disrupting cyclin D1‐CDK6 complex formation, although further mechanistic studies are needed. Overall, the study demonstrates that 4‐PBA‐induced downregulation of ER‐stress responsive genes leads to reduced inflammation and cell proliferation in colon cancer cells, underscoring the interconnected roles of ER‐stress, inflammation, and tumor progression.

Evidence suggests that IL‐6 is a key cytokine in chronic colitis, and reduced IL‐10 activity leads to increased levels of IL‐6, IFN‐γ, and TNF [45, 46, 47]. IFN‐γ, in particular, is elevated in inflammatory diseases like colon cancer [47]. Consistent with these findings, this study observed elevated IL‐6 and IFN‐γ levels in untreated Caco‐2 cells, which were significantly reduced following 72 h of 4‐PBA treatment. Additionally, a decrease in CXCL10 levels was noted in 4‐PBA‐treated groups, aligning with the observed reduction in IFN‐γ. These results highlight 4‐PBA's potential to downregulate pro‐inflammatory cytokines, suggesting a link between ER‐stress reduction and suppression of inflammatory responses in colon cancer cells.

The results of the study indicated that treatment with 1 mM 4‐PBA significantly increased catalase expression, while lower doses (500 and 750 μM) showed no notable effect. This suggests that 4‐PBA helps counteract oxidative stress, possibly by restoring catalase levels, consistent with previous findings [48]. ER‐stress has been linked to cytochrome‐C release from mitochondria, which elevates ROS through enhanced ubi‐semiquinone radical formation [49, 50]. Accumulation of ER‐stress and ROS can increase inflammation and may deteriorate cancer progression [51, 52]. Correspondingly, this study found reduced ROS levels in 4‐PBA‐treated cells compared to controls, indicating the compound's ability to neutralize oxidative stress. Lower ROS levels support the function of antioxidant enzymes with anti‐inflammatory properties. Given that cancer cells typically maintain high ROS levels due to metabolic demands, disrupting this redox balance through treatments like 4‐PBA can impair cancer cell survival [53].

The observed increase in cell senescence in Caco‐2 cells following 4‐PBA treatment may be attributed to its ability to reduce ER‐stress. By alleviating ER‐stress, 4‐PBA could create a cellular environment that promotes senescence. However, further research is needed to clarify the specific mechanisms through which 4‐PBA regulates the interplay between ER‐stress, senescence, and inflammation in these cells.

Although the current study has certain limitations, particularly the absence of protein‐level validation for UPR marker alterations, ER‐stress gene expressions was quantified using qPCR, and alterations in inflammatory cytokine proteins were confirmed. However, additional studies involving protein‐level analysis, such as Western blotting, to evaluate ER‐stress proteins could not be conducted. Future research should incorporate comprehensive protein‐level assessments of PERK, ATF6, IRE1α, CHOP, and associated effectors to reinforce the in silico and in vitro findings and to elucidate the modulatory effects of 4‐PBA on ER‐stress within colon cancer models.

## 6 Conclusion

This study demonstrated that 4‐PBA exhibits anti‐inflammatory and anti‐proliferative effects, potentially through the downregulation of ER‐stress genes, cell cycle‐related genes, and the regulation of inflammation in colon cancer cell lines. These findings are further supported by in silico studies, which suggest that 4‐PBA significantly influences the conformational properties of proteins associated with ER‐stress. However, further in‐depth study on the role of 4‐PBA is necessary, both in vitro with more cell lines and in vivo animal models.

## Author Contributions

A.B. and A.K.D. designed and conceptualized the work. D.D. and A.D. performed the experiments and wrote the manuscript. N.B. and S.N.R. performed molecular docking and molecular dynamics simulation. A.K.D., A.B., and S.P. critically reviewed and edited the complete manuscript.

## Ethics Statement

The authors have nothing to report.

## Conflicts of Interest

The authors declare no conflicts of interest.

## Supporting information


**Data S1:** Supporting Information.

**Table S2:** Table showing the list of negative control residues of ER‐stress markers with 4‐PBA.

## Data Availability

The data that support the findings of this study are available from the corresponding author upon reasonable request.
