# Supplementary material for: 4‐Phenylbutyric Acid Reduces the Proliferation in Colon Cancer Cell Lines Through Modulating the Cell Cycle Regulatory Genes: An In Silico and In Vitro Approach
Source: Cancer Rep (Hoboken). 2025 Sep 15;8(9):e70352. doi: 10.1002/cnr2.70352 (PMC12435982; doi:10.1002/cnr2.70352)
Supplement: Supplementary file 1 — Data S1: Supporting Information. [file CNR2-8-e70352-s001.pdf]

NATIONAL CENTRE FOR CELL SCIENCE, PUNE
राष्ट्रीय कोशिका विज्ञान केन्द्र, पुणे

Cell Line Authentication Report

Job No: 02|2022-23, PN:- 39

Name of Cell Line: Caco-2

Methodology:
Sixteen short tandem repeat (STR) loci were amplified using commercially available AmpFISTR® Identifiler® Plus PCR Amplification Kit from Applied Bio systems. The cell line sample was processed using the Applied Bio systems® 3500 Genetic Analyser. Data was analysed using Gene Mapper® ID-X v1.5 software (Applied Biosystems). Appropriate positive and negative controls were used and confirmed for each sample.

STR Profile:

D5S818:	12, 13
D7S820:	11, 12
D13S317:	11, 13, 14
D16S539:	12, 13
CSF1PO:	11
TH01:	6
TPOX:	9, 11
vWA:	16, 18
Amelogenin:	X
D21S11:	30
D81179:	12, 14
D3S1358:	14, 17
D2S1338:	17, 25
D19S433:	15
D18S51:	12
FGA:	19

Percent match between tested cell line sample and ATCC STR profile database: 100%

PNagvenkar
19-04-2022

Dr. Punam Nagvenkar
Scientist D
Cell Repository

CELL REPOSITORY
National Centre for Cell Science,
S.P. Pune University Campus,
Ganeshkhind, Pune - 411 007.

NATIONAL CENTRE FOR CELL SCIENCE, PUNE
राष्ट्रीय कोशिका विज्ञान केन्द्र, पुणे

Cell Line Authentication Report

Job No: 15

***Name of Cell Line*:** HCT-116 P26

***Methodology*:**
Sixteen short tandem repeat (STR) loci were amplified using commercially available AmpFlSTR® Identifiler® Plus PCR Amplification Kit from Applied Bio systems. The cell line sample was processed using the Applied Bio systems® 3500 Genetic Analyser. Data was analysed using Gene Mapper® ID-X v1.5 software (Applied Biosystems). Appropriate positive and negative controls were used and confirmed for each sample.

***STR Profile*:**

D5S818:	10, 11
D7S820:	11, 12
D13S317:	10, 12
D16S539:	11, 13
CSF1PO:	7, 10
TH01:	8, 9
TPOX:	8, 9
vWA:	17, 22
Amelogenin:	X, Y
D21S11:	29, 30
D81179:	11, 12, 14
D3S1358:	12, 18, 19
D2S1338:	16
D19S433:	12, 13
D18S51:	16, 17
FGA:	18, 23

Percent match between tested cell line sample and ATCC STR profile database: 100%

CELL REPOSITORY
National Centre for Cell Science,
S.P. Pune University Campus,
Ganeshkhind, Pune - 411 007.

NATIONAL CENTRE FOR CELL SCIENCE, PUNE
राष्ट्रीय कोशिका विज्ञान केन्द्र, पुणे

Cell Line Authentication Report

Job No: 16

***Name of Cell Line*:** SW-620 PN14

***Methodology*:**
Sixteen short tandem repeat (STR) loci were amplified using commercially available AmpFISTR® Identifiler® Plus PCR Amplification Kit from Applied Bio systems. The cell line sample was processed using the Applied Bio systems® 3500 Genetic Analyser. Data was analysed using Gene Mapper® ID-X v1.5 software (Applied Biosystems). Appropriate positive and negative controls were used and confirmed for each sample.

***STR Profile*:**

D5S818:	13
D7S820:	8, 9
D13S317:	12
D16S539:	9, 13
CSF1PO:	13, 14
TH01:	8
TPOX:	11
vWA:	16
Amelogenin:	X
D21S11:	30, 30.2
D81179:	13
D3S1358:	16
D2S1338:	17, 24
D19S433:	13
D18S51:	13
FGA:	24

Percent match between tested cell line sample and ATCC STR profile database: 100%

CELL REPOSITORY
National Centre for Cell Science,
S.P. Pune University Campus,
Ganeshkhind, Pune - 411 007.

NATIONAL CENTRE FOR CELL SCIENCE, PUNE
राष्ट्रीय कोशिका विज्ञान केन्द्र, पुणे

Cell Line Authentication Report

Job No: 16

Name of Cell Line: SW-480 P34

Methodology:
Sixteen short tandem repeat (STR) loci were amplified using commercially available AmpFISTR® Identifiler® Plus PCR Amplification Kit from Applied Bio systems. The cell line sample was processed using the Applied Bio systems® 3500 Genetic Analyser. Data was analysed using Gene Mapper® ID-X v1.5 software (Applied Biosystems). Appropriate positive and negative controls were used and confirmed for each sample.

STR Profile:

D5S818:	13
D7S820:	8
D13S317:	12
D16S539:	13
CSF1PO:	13, 14
TH01:	8
TPOX:	11
vWA:	16
Amelogenin:	X
D21S11:	30, 30.2
D81179:	13
D3S1358:	15
D2S1338:	17, 24
D19S433:	13
D18S51:	13
FGA:	24

Percent match between tested cell line sample and ATCC STR profile database: 100%

CELL REPOSITORY
National Centre for Cell Science,
S.P. Pune University Campus,
Ganeshkhind, Pune - 411 007.
