# Supplementary material for: 4‐Phenylbutyric Acid Reduces the Proliferation in Colon Cancer Cell Lines Through Modulating the Cell Cycle Regulatory Genes: An In Silico and In Vitro Approach
Source: Cancer Rep (Hoboken). 2025 Sep 15;8(9):e70352. doi: 10.1002/cnr2.70352 (PMC12435982; doi:10.1002/cnr2.70352)
Supplement: Supplementary file 2 — Table S2: Table showing the list of negative control residues of ER‐stress markers with 4‐PBA. [file CNR2-8-e70352-s002.pdf]

Supplementary Table 2: Table showing the list of negative control residues of ER-stress markers with 4-PBA

ER-Stress marker	PDB-ID	Negative Control Residues
**PDI**	2BJX	ALA119 ALA120 THR121 THR122 LEU123 PRO124 ASP125 GLY126 ALA127 ALA128 ALA129 GLU130 SER131 LEU132 VAL133 GLU134 SER135 SER136 GLU137 VAL138 ALA139 VAL140 ILE141 GLY142 PHE143 PHE144 LYS145 ASP146 VAL147 GLU148 SER149 ASP150 SER151 ALA152 LYS153 GLN154 PHE155 LEU156 GLN157 ALA158 ALA159 GLU160 ALA61 ILE162 ASP163 ASP164 ILE165 PRO166 PHE167 GLY168 ILE169 THR170 SER171 ASN172 SER173 ASP174 VAL175 PHE176 LYS 178 LYS178 GLN180 ASP182 LYS183 ASP184 VAL187 LEU188 PHE189 LYS190 LYS191 PHE192 ASP193 GLU194 GLY195 GLU200 GLY201 GLU202 VAL203 THR204 LYS205 GLU206 ASN207 LEU208 LEU209 ASP210 PHE211 ILE212 LYS213 HIS214 GLN216 LEU217 PRO218 LEU219 VAL220 ILE221 GLU222 PHE223 THR224 GLU225 GLN226 THR227
**IRE1-α luminal Domain**	2HZ6	PRO29 GLU30 THR31 LEU32 LEU33 PHE34 VAL35 THR37 LEU38 ASP39 SER41 LEU42 HIS43 ALA44 VAL45 SER46 LYS47 ARG48 THR49 GLY50 SER51 ILE52 LYS53 TRP54 THR55 LEU56 LYS57 PRO60 LEU62 GLN63 VAL64 PRO65 PRO71 ALA72 PHE73 LEU74 PRO75 ASP76 ASN78 ASP79 GLY80 SER81 LEU82 TYR83 THR84 LEU85 GLU91 GLY92 LEU93 THR94 LYS95 LEU96 PRO97 PHE98 THR99 ILE100 PRO101 GLU102 LEU103 VAL104 GLN105 ALA106 SER107 PRO108 CYS109 ARG110 LEU116 TYR117 MET118 GLY119

		LYS120 LYS121 GLN122 ASP123 ILE124 TRP125 TYR126 VAL127 ILE128 ASP129 LEU130 LEU153 LEU154 TYR155 LEU156 GLY157 THR159 GLU160 TYR161 THR162 ILE163 THR164 MET165 TYR166 ASP167 THR168 LYS169 THR170 ARG171 GLU172 LEU173 ARG174 TRP175 ASN176 ALA177 THR178 TYR182 ALA183 ALA184 SER185 LEU186 PRO187 GLU188 ASP189 ASP190 VAL191 ASP192 TYR193 LYS194 MET195 SER196 HIS197 PHE198 VAL199 SER200 ASN201 GLY202 ASP203 GLY204 LEU205 VAL206 VAL207 THR208 VAL209 ASP210 SER211 GLU212 SER213 GLY214 ASP215 VAL216 LEU217 TRP218 ILE219 GLN220 ASN221 TYR222 ALA223 SER224 PRO225 VAL226 PHE229 VAL231 TRP232 GLN233 ARG234 GLU235 GLY236 LEU237 ARG238 LYS239 VAL240 MET241 HIS242 ILE243 ASN244 VAL245 ALA246 VAL247 GLU248 THR249 LEU250 ARG251 TYR252 LEU253 THR254 PHE255 MET256 SER257 GLY258 GLU259 VAL260 GLY261 ARG262 ILE263 THR264 LYS265 TRP266 LYS267 TYR268 PRO269 PHE270 PRO271 LYS272 GLU273 THR274 GLU275 ALA276 LYS277 SER278 LYS279 LEU280 THR281 PRO282 THR283 LEU284 TYR285 VAL286 GLY287 LYS288 TYR289 SER290 THR291 SER292 LEU293 TYR294 ALA295 SER296 PRO297 SER298 MET299 VAL300 HIS301 GLU302 GLY303 VAL304 ALA305 VAL306 VAL307 TYR358 TRP359 LEU360 LEU361 ILE362 GLY363 HIS364 HIS365 GLU366 THR367 PRO368
**NRF2**	2LZ1	MET1 GLY2 HIS3 HIS4 HIS5 HIS6 HIS7 HIS8 SER9 HIS10 MET11 LYS 12 HIS13 SER14 SER15 LEU17 ALA19 THR22 ARG23 ASP24 LEU26 ARG27 ALA28 LYS29 ALA30 LEU31

		HIS32 ILE33 PRO34 PHE35 PRO36 LYS39 ILE40 ASN42 LEU43 PRO44 VAL45 VAL46 ASP47 PHE48 ASN49 GLU50 MET51 MET52 SER53 LYS54 GLU55 GLN56 PHE57 ASN58 GLU59 ALA60 GLN61 LEU62 ALA63 LEU64 ILE65 ARG66 ASP67 ILE68 ARG69 ARG70 ARG71 GLY72 LYS73 ASN74 LYS75 ALA77 ALA78 GLN79 ASN80 CYS81 ARG82 LYS83 ARG84 LYS85 GLU87 ASN88 ILE89 VAL90
**GRP78**	3LDN	ASP 26 VAL 27 GLY 28 THR29 VAL30 VAL31 GLY32 ILE33 LEU35 GLY36 SER40 CYS41 VAL42 GLY43 VAL44 PHE45 LYS46 ASN47 GLY48 ARG49 VAL50 GLU51 ILE52 ILE53 ALA54 ASN55 ASP56 GLN57 GLY58 ASN59 ARG60 ILE61 THR62 PRO63 SER64 TYR65 VAL66 ALA67 PHE68 THR69 PRO70 GLU71 GLY72 GLU73 ARG74 LEU75 ILE76 GLY77 ASP78 ALA79 ALA80 LYS81 ASN82 GLN83 LEU84 THR85 SER86 ASN87 PRO88 GLU89 ASN90 THR91 VAL92 PHE93 ASP94 ALA95 LYS96 ARG97 LEU98 ILE99 GLY100 ARG101 THR102 TRP103 ASN104 ASP105 PRO106 SER107 VAL108 GLN109 GLN110 ASP111 ILE112 LYS113 PHE114 LEU115 PRO116 PHE117 LYS118 VAL119 VAL120 GLU121 LYS122 LYS123 THR124 LYS125 PRO126 TYR127 ILE128 GLN129 VAL130 ASP131 ILE132 GLY133 GLY134 GLY135 GLN136 THR137 LYS138 THR139 PHE140 ALA141 PRO142 GLU143 GLU144 ILE145 SER146 ALA147 MET148 VAL149 LEU150 THR151 LYS152 MET153 LYS154 GLU155 THR156 ALA157 GLU158 ALA159 TYR160 LEU161 GLY162 LYS163 LYS164 VAL165 THR166 HIS167 ALA168 VAL169 VAL170 THR171 VAL172 PRO173 ALA174 TYR175 PHE176 ASN177 ASP178 ALA179 GLN180 ARG181 GLN182 ALA183 THR184 LYS185

		ASP186 ALA187 GLY188 THR189 ILE190 ALA191 GLY192 LEU193 ASN194 VAL195 MET196 ARG197 ILE198 ILE199 ASN200 GLU201 PRO202 THR203 ALA204 ALA205 ALA206 ILE207 ALA208 TYR209 GLY210 LEU211 ASP212 LYS213 ARG214 GLU215 GLY216 GLU217 LYS218 ASN219 ILE220 LEU221 VAL222 PHE223 ASP224 LEU225 GLY228 PHE230 ASP231 VAL232 SER233 LEU234 LEU235 THR236 ILE237 ASP238 ASN239 GLY240 VAL241 PHE242 GLU243 VAL244 VAL245 ALA246 THR247 ASN248 GLY249 ASP250 THR251 HIS252 LEU253 GLY254 GLY255 GLU256 ASP257 PHE258 ASP259 GLN260 ARG261 VAL262 MET263 GLU264 HIS265 PHE266 ILE267 LYS268 LEU269 TYR270 LYS271 LYS272 LYS273 THR274 GLY275 LYS276 ASP277 VAL278 ARG279 LYS280 ASP281 ASN282 ARG283 ALA284 VAL285 GLN286 LYS287 LEU288 ARG289 ARG290 GLU291 VAL292 GLU293 LYS294 ALA295 LYS296 ARG297 ALA298 LEU299 SER300 SER301 GLN302 HIS303 GLN304 ALA305 ARG306 ILE307 GLU308 ILE309 GLU310 SER311 PHE312 TYR313 GLU314 GLY315 GLU316 ASP317 PHE318 SER319 GLU320 THR321 LEU322 THR323 ARG324 ALA325 LYS326 PHE327 GLU328 GLU329 LEU330 ASN331 MET332 ASP333 LEU334 PHE335 ARG336 SER337 THR338 MET339 LYS340 PRO341 VAL342 GLN343 LYS344 VAL345 LEU346 GLU347 ASP348 SER349 ASP350 LEU351 LYS352 LYS353 SER354 ASP355 ILE356 ASP357 GLU358 ILE359 VAL360 LEU361 VAL362 GLY364 SER365 THR366 ARG367 ILE368 PRO369 LYS370 ILE371 GLN372 GLN373 LEU374 VAL375 LYS376 GLU377 PHE378 PHE379 ASN380 GLY381 LYS382 GLU383 PRO384 SER385

		ARG386 GLY387 ILE388 ASN389 GLU392 ALA393 VAL394 ALA395 TYR396 GLY397 ALA398 ALA399 VAL400 GLN401 ALA402 GLY403 VAL404 LEU405 SER406 GLY407
**PERK**	4G34	GLY586 ARG587 TYR588 LEU589 THR590 ASP591 PHE592 GLU593 PRO594 ILE595 GLN596 CYS597 GLY599 ARG600 GLY601 GLY602 PHE603 GLY604 VAL605 PHE607 GLU608 ALA609 LYS610 ASN611 LYS612 VAL613 ASP614 ASP615 CYS616 ASN617 TYR618 ALA619 ILE620 LYS621 ARG622 ILE623 ARG624 LEU625 PRO626 ASN627 ARG628 GLU629 LEU630 ALA631 ARG632 GLU633 LYS634 VAL635 MET636 ARG637 GLU638 VAL639 LYS640 ALA641 LEU642 ALA643 LYS644 LEU645 GLU646 HIS647 PRO648 GLY649 ILE650 ARG652 TYR653 PHE654 ASN655 ALA656 TRP657 LEU658 GLU659 LYS868 ASN869 LYS880 VAL881 TYR882 LEU883 TYR884 ILE885 GLN886 GLN888 LEU889 ARG891 LYS892 GLU893 ASN894 LEU895 LYS896 ASP897 TRP898 MET899 ASN900 GLY901 ARG902 CYS903 THR904 ILE905 GLU906 GLU907 ARG908 GLU909 ARG910 SER911 VAL912 CYS913 LEU914 HIS915 ILE916 PHE917 LEU918 GLN919 ILE920 ALA921 GLU922 ALA923 VAL924 GLU925 PHE926 LEU927 HIS928 SER929 LYS930 GLY931 LEU932 MET933 HIS934 ARG935 ASP936 LEU937 LYS938 PRO939 SER940 ASN941 ILE942 PHE944 THR945 MET946 AASP947 ASP948 VAL949 VAL950 LYS951 VAL952 PHE955 GLY956 LEU957 GLY985 THR986 LYS987 LEU988 TYR989 MET990 SER991 PRO992 GLU993 GLN994 ILE995 HIS996 GLY997 ASN998 SER999 TYR1000 SER1001 HIS1002 LYS1003 VAL1004 ASP1005 ILE1006 PHE1007 SER1008 LEU1009 GLY1010 LEU1011

		ILE1012 LEU1013 PHE1014 GLU1015 LEU1016 LEU1017 TYR1018 PRO1019 PHE1020 SER1021 THR1022 GLN1023 MET1024 GLU1025 ARG1026 VAL1027 ARG1028 THR1029 LEU1030 THR1031 ASP1032 VAL1033 ARG1034 ASN1035 LEU1036 LYS1037 PHE1038 PRO1039 PRO1040 LEU1041 PHE1042 THR1043 AGLN1044 LYS1045 TYR1046 PRO1047 CYS1048 GLU1049 TYR1050 VAL1051 MET1052 VAL1053 GLN1054 ASP1055 MET1056 LEU1057 SER1058 PRO1059 SER1060 PRO1061 MET1062 GLU1063 ARG1064 PRO1065 GLU1066 ALA1067 ILE1068 AASN1069 ILE1070 ILE1071 GLU1072 ASN1073 ALA1074 VAL1075 PHE1076 GLU1077 ASP1078
**IRE1-α**	5HGI	SER562 VAL563 VAL564 ILE565 VAL566 GLY567 LYS568 ILE569 SER570 PHE571 CYS572 PRO573 LYS574 ASP575 VAL576 LEU577 GLY578 HIS579 GLY583 THR584 ILE585 VAL586 TYR587 ARG588 GLY589 MET590 PHE591 ASP592 ASN593 ARG594 ASP595 VAL596 ALA597 VAL598 LYS599 ARG600 ILE601 LEU602 PRO603 GLU604 CYS605 PHE606 SER607 PHE608 ALA609 ASP610 ARG611 GLU612 VAL613 GLN614 LEU615 LEU616 ARG617 GLU618 SER619 ASP620 GLU621 HIS622 PRO623 ASN624 VAL625 ILE626 ARG627 TYR628 PHE629 CYS630 THR631 GLU632 ARG635 GLN636 PHE637 GLN638 TYR639 ILE640 ALA641 ILE642 GLU643 LEU644 CYS645 ALA646 ALA647 THR648 LEU649 GLN650 GLU651 TYR652 VAL653 GLU654 GLN655 LYS656 ASP657 PHE658 ALA659 HIS660 LEU661 GLY662 LEU663 GLU664 PRO665 THR667 LEU668 LEU669 GLN670 GLN671 THR672 THR673 SER674 GLY675 LEU676 ALA677 HIS678 LEU679

		HIS680 SER681 LEU682 ASN683 ILE684 VAL685 HIS686 ARG687 ASP688 LEU689 LYS690 PRO691 HIS692 ASN693 ILE694 LEU695 ILE696 SER697 MET698 PRO699 ASN700 ALA701 HIS702 GLY703 LYS704 ILE705 LYS706 ALA707 MET708 ILE709 SER710 ASP711 PHE712 GLY713 LEU714 CYS715 LYS716 LYS717 LEU718 ALA719 VAL720 GLY721 ARG722 HIS723 SER724 PHE725 SER726 ARG727 ARG728 SER729 GLY730 VAL731 PRO732 GLY733 THR734 GLU735 GLY736 TRP737 ILE738 ALA739 PRO740 GLU741 MET742 LEU743 SER744 GLU745 ASP746 CYS747 LYS748 GLU749 ASN750 PRO751 THR752 TYR753 THR754 VAL755 ASP756 ILE757 PHE758 SER759 ALA760 GLY761 CYS762 VAL763 PHE764 TYR765 TYR766 VAL767 ILE768 SER769 GLU770 GLY771 SER772 HIS773 PRO774 PHE775 GLY776 LYS777 SER778 LEU779 GLN780 ARG781 GLN782 ALA783 ASN784 ILE785 LEU786 LEU787 GLY788 ALA789 CYS790 SER791 LEU792 ASP793 CYS794 LEU795 HIS796 PRO797 GLU798 LYS799 HIS800 ASP802 VAL803 ALA805 ARG806 GLU807 LEU808 ILE809 GLU810 LYS811 MET812 ILE813 ALA814 MET815 ASP816 PRO817 GLN818 LYS819 ARG820 PRO821 SER822 ALA823 LYS824 HIS825 VAL826 LEU827 LYS828 HIS829 SER834 LEU835 GLU836 LYS837 LEU839 GLN840 PHE841 PHE842 GLN843 ASP844 VAL845 SER846 ASP847 ARG848 ILE849 GLU850 LYS851 GLU852 SER853 LEU854 ASP855 GLY856 PRO857 ILE858 VAL859 LYS860 GLN861 LEU862 GLU863 ARG864 GLY865 GLY866 ARG867 ALA868 VAL869 VAL870 LYS871 MET872 ASP873 TRP874 ARG875 GLU876 ASN877 ILE878 THR879 VAL880 PRO881 LEU882

		GLN883 THR884 ASP885 LEU886 ARG890 THR891 TYR892 LYS893 GLY894 GLY895 SER896 VAL897 ARG898 ASP899 LEU900 LEU901 ARG902 ALA903 MET904 ARG905 ASN906 LYS907 LYS908 HIS909 HIS910 TYR911 ARG912 GLU913 LEU914 PRO915 ALA916 GLU917 VAL918 ARG919 GLU920 THR921 LEU922 GLY923 SER924 LEU925 PRO926 ASP927 ASP928 PHE929 VAL930 CYS931 TYR932 PHE933 THR934 SER935 ARG936 PHE937 PRO938 HIS939 LEU940 LEU941 ALA942 HIS943 THR944 TYR945 ARG946 ALA947 MET948 GLU949 LEU950 CYS951 SER952 HIS953 GLU954 ARG955 LEU956 PHE957 GLN958 PRO959 TYR960 TYR961 PHE962 HIS963 GLU964
